# The Nonphysiological Reductant Sodium Dithionite and [FeFe] Hydrogenase: Influence on the Enzyme Mechanism

**DOI:** 10.1021/jacs.1c07322

**Published:** 2021-10-20

**Authors:** Maria Alessandra Martini, Olaf Rüdiger, Nina Breuer, Birgit Nöring, Serena DeBeer, Patricia Rodríguez-Maciá, James A. Birrell

**Affiliations:** † Department of Inorganic Spectroscopy, Max Planck Institute for Chemical Energy Conversion, Stiftstraße 34-36, 45470 Mülheim an der Ruhr, Germany; ‡ Inorganic Chemistry Laboratory, Department of Chemistry, University of Oxford, South Parks Road, Oxford, OX1 3QR, U.K.

## Abstract

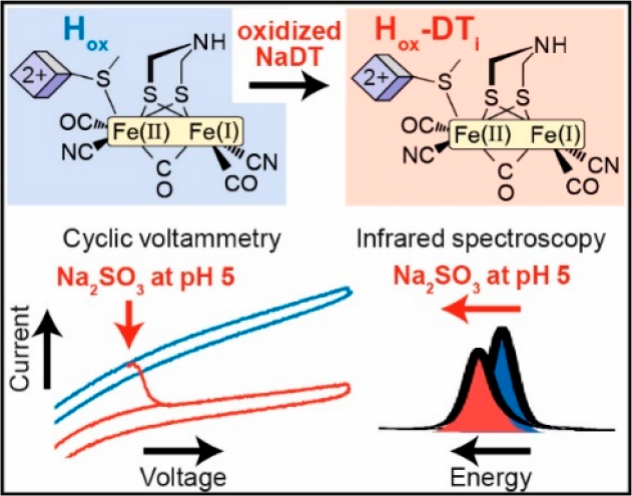

[FeFe] hydrogenases are highly active enzymes for interconverting protons and electrons with hydrogen (H_2_). Their active site H-cluster is formed of a canonical [4Fe-4S] cluster ([4Fe-4S]_H_) covalently attached to a unique [2Fe] subcluster ([2Fe]_H_), where both sites are redox active. Heterolytic splitting and formation of H_2_ takes place at [2Fe]_H_, while [4Fe-4S]_H_ stores electrons. The detailed catalytic mechanism of these enzymes is under intense investigation, with two dominant models existing in the literature. In one model, an alternative form of the active oxidized state H_ox_, named H_ox_H, which forms at low pH in the presence of the nonphysiological reductant sodium dithionite (NaDT), is believed to play a crucial role. H_ox_H was previously suggested to have a protonated [4Fe-4S]_H_. Here, we show that H_ox_H forms by simple addition of sodium sulfite (Na_2_SO_3_, the dominant oxidation product of NaDT) at low pH. The low pH requirement indicates that sulfur dioxide (SO_2_) is the species involved. Spectroscopy supports binding at or near [4Fe-4S]_H_, causing its redox potential to increase by ∼60 mV. This potential shift detunes the redox potentials of the subclusters of the H-cluster, lowering activity, as shown in protein film electrochemistry (PFE). Together, these results indicate that H_ox_H and its one-electron reduced counterpart H_red_′H are artifacts of using a nonphysiological reductant, and not crucial catalytic intermediates. We propose renaming these states as the “dithionite (DT) inhibited” states H_ox_-DT_i_ and H_red_-DT_i_. The broader potential implications of using a nonphysiological reductant in spectroscopic and mechanistic studies of enzymes are highlighted.

## Introduction

**Figure 1:**
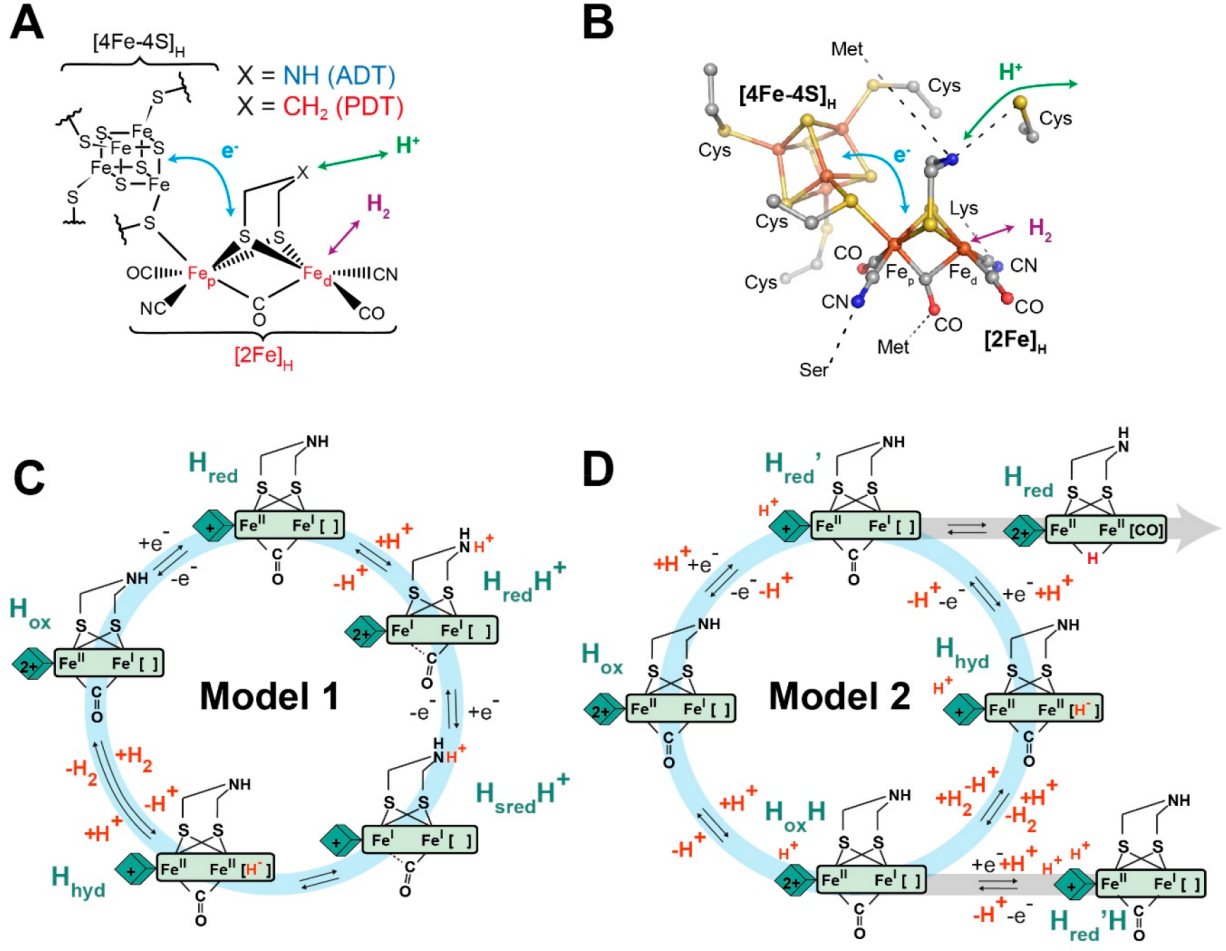
Structure of the H-cluster and proposed catalytic cycles. (A) Schematic showing the chemical structure of the [2Fe]_H_ subcluster attached covalently to the [4Fe-4S]_H_ subcluster. “X” in the bridgehead position is an NH group in the native ADT ligand and is CH_2_ in the synthetic chemical variant PDT. (B) Structure of [2Fe]_H_ and [4Fe-4S]_H_ from HydA1 from *Clostridium pasteurianum* (PDB: 4XDC(26)) showing the nearby amino acids that interact with [2Fe]_H_. (C) Catalytic cycle Model 1 in which one-electron reduction of [4Fe-4S]_H_ is followed by proton-coupled electronic rearrangement to give the H_red_H^+^ state.^14^ A further one-electron reduction of [4Fe-4S]_H_ gives the H_sred_H^+^ state,^16^ which is then followed by rearrangement to give the H_hyd_ state with a terminal hydride at Fe_d_.^19−21^ The subsequent steps leading to H_2_ formation are not shown. (D) Catalytic cycle Model 2 in which proton-coupled electron transfer at [4Fe-4S]_H_ converts H_ox_ to H_red_′, which can engage in further proton-coupled electron transfer to give the terminal hydride-containing H_hyd_ state.^10,19^ H_hyd_ then reacts with an additional proton to generate H_2_ leaving a protonated H_ox_H state.^27^ Alternatively, H_red_′ can rearrange to give a less active H_red_ state containing a bridging hydride,^28^ which proceeds through a low activity pathway. H_ox_H appears to undergo one-electron reduction to H_red_′H, but this is not included in the catalytic cycle.^10,27^

[FeFe] hydrogenases are highly active metalloenzymes that catalyze the reversible reduction of protons to molecular hydrogen.^1,2^ Their active site, the H-cluster, comprises a unique diiron subcluster ([2Fe]_H_) and a canonical [4Fe-4S] subcluster ([4Fe-4S]_H_), covalently linked by a cysteine thiolate^3,4^ (Figure 1 A and B). The Fe of [2Fe]_H_ that is closest to [4Fe-4S]_H_ is known as the proximal Fe (Fe_p_), while the Fe furthest from the cluster is known as the distal Fe (Fe_d_). In [2Fe]_H_, the Fe ions are coordinated by two terminal CN^–^ and two terminal CO ligands (one on each Fe), a bridging CO, and a bridging 2-azapropane-1,3-dithiolate (ADT) ligand.^5,6^ During H_2_ conversion, the H-cluster goes through a series of redox transitions, where the Fe ions change oxidation states, as well as protonation/deprotonation steps.^7−9^ While several catalytic intermediate states have been well characterized with a variety of spectroscopic techniques, structural models based on X-ray diffraction data on crystals in spectroscopically defined states are not generally available. Thus, in the absence of structural models supported by experimental data, computational chemistry has played an important role in proposing likely structures of the active site in the catalytic intermediates based on spectroscopic data. However, divergent results from various groups have led to several possible models of the catalytic cycle of [FeFe] hydrogenases.^8,10−12^ These can be summarized in two main models (here referred to as Model 1 and 2, Figure 1C and D respectively).

The most oxidized state of the active enzyme, H_ox_, is generally accepted to be the starting point of the catalytic cycle and has a mixed valence of Fe_p_(II)Fe_d_(I) in [2Fe]_H_,^13^ and an oxidized [4Fe-4S]_H_^2+^. In Model 1 (Figure 1C), one-electron reduction of H_ox_ is proposed to yield two possible states H_red_ and H_red_H^+^ (in our nomenclature), whose relative population depends on the pH. In H_red_, the electron is thought to be localized preferentially on [4Fe-4S]_H_. In H_red_H^+^, the electron is thought to be transferred to the [2Fe]_H_ subcluster (with an Fe_p_(I)Fe_d_(I) configuration) and a proton (from the proton transfer pathway) to bind to the nitrogen in the ADT bridge giving an NH_2_^+^.^14,15^ This process of proton-coupled electronic rearrangement (PCER) of the H-cluster is a crucial component of Model 1. A further one-electron reduction of H_red_H^+^ yields the H_sred_H^+^ state with a reduced [4Fe-4S]_H_^+^.^16,17^ The protonated ADT ligand in both H_red_H^+^ and H_sred_H^+^ appears to be able to transfer the proton to Fe_d_ generating an Fe-bound hydride in the H_hyd_ state.^18−23^ Finally, the H_hyd_ state is thought to gain an additional proton, which may trigger a similar PCER process as in the H_red_ state, and form a H_2_ molecule bound to Fe_d_, which can then leave the enzyme via a hydrophobic gas channel.^24^ Recently, photoexcitation of H_red_H^+^ and H_sred_H^+^ was shown to generate two different forms of H_hyd_, known as H_hyd:ox_ and H_hyd:red_, where the former has an oxidized [4Fe-4S]_H_ and the latter has a reduced [4Fe-4S]_H_.^25^

In Model 2 (Figure 1D),^8^ the H_red_ state (referred to as H_red_′) is formed from H_ox_ by proton-coupled electron transfer (PCET) at [4Fe-4S]_H_. This was suggested based on IR spectroelectrochemical titrations at various pH values that showed a pH-dependent redox potential of [4Fe-4S]_H_.^10^ Specifically, the proton is thought to bind to one of the cysteine ligands coordinating the cluster. This state then undergoes an additional PCET at [2Fe]_H_ to give the H_hyd_ state,^19^ and the proton is retained on [4Fe-4S]_H_. Hydrogen is then formed by additional protonation leaving an oxidized H-cluster but still protonated at [4Fe-4S]_H_, a state called H_ox_H.^27^ In Model 2, H_red_H^+^ (referred to as H_red_) and H_sred_H^+^ (not shown in Figure 1D) contain a bridging hydride (μH^–^) and an apical CO ligand,^28^ and are considered to be part of a low activity pathway. Lastly, reduction of H_ox_H to H_red_′H has also been observed,^10^ but its place in the catalytic cycle remains to be determined.

The H_ox_H and H_red_′H states in Model 2 have been reported to accumulate at low pH only in the presence of sodium dithionite (NaDT) (see Supporting Information for further details).^10,27^ NaDT (Na_2_S_2_O_4_, also sodium hydrosulfite) is widely used in biochemistry as an oxygen scavenger and low potential reducing agent (*E*^0^*’* = −0.66 V vs SHE at pH 7 and 25 °C).^29^ For example, it is commonly employed to protect metalloproteins from oxidative damage caused by trace amounts of oxygen during purification and handling, or to poise metallocofactors in reduced states for their characterization. However, one of the pitfalls of its use is the failure to consider that NaDT and its oxidation products can engage in side-reactions with the system under study. Several studies on sulfite-reducing enzymes have highlighted how oxidation of NaDT can be a significant source of SO_3_^2–^, the substrate for these enzymes, which can bind to the active site and complicate the interpretation of spectroscopic studies and activity measurements.^30−32^ In a recent report, during the semisynthetic assembly of the FeMo cofactor of nitrogenase, the donor of the ninth sulfur ligand was found to be the SO_3_^2–^ generated by the oxidation or degradation of NaDT present in the assay.^33^ Numerous studies have reported the interaction of oxidation products of NaDT with various enzymes including nitrite reductase,^34−36^ DMSO reductase,^37^ monomethylamine methyltransferase,^38^ acetyl CoA synthase,^39^ and formation of adducts to flavins^40−42^ and cobalamin.^43,44^ Additionally, the slow dissociation of NaDT into SO_2_^•–^ radicals (the active reducing species) has been shown to be problematic in mechanistic studies of nitrogenase.^45^

In light of the dependence of H_ox_H and H_red_′H on NaDT, and of NaDT’s reported “non-innocent” behavior, we decided to investigate the effect of NaDT and its oxidation products on [FeFe] hydrogenases. Formation of the H_ox_H state was observed when the [FeFe] hydrogenase from *Chlamydomonas reinhardtii* (*Cr*HydA1) was treated with oxidized NaDT. Addition of Na_2_SO_3_ (the dominant oxidation product of NaDT^46^) to *Cr*HydA1 at low pH reproduced the same effect as oxidized NaDT. Under H_2_, H_red_′H was also observed. We propose that, at low pH, the dissolved sulfur dioxide (SO_2_) generated by the protonation of SO_3_^2–^ binds to the H-cluster. Based on our spectroscopic observations, we hypothesize that this occurs near [4Fe-4S]_H_ with submicromolar binding affinity as estimated by IR titrations. Based on the ratios of the H_ox_/H_red_ and H_ox_H/H_red_′H states under H_2_, binding of SO_2_ causes the redox potential of [4Fe-4S]_H_ to increase by ∼60 mV. The effect of this on catalysis was investigated via protein film electrochemistry (PFE), showing that binding of SO_2_ has an inhibitory effect on both H^+^ reduction and H_2_ oxidation activity of [FeFe] hydrogenases. Together, these results reveal that the so-called H_ox_H and H_red_′H states are not related to protonation events at the [4Fe-4S]_H_ subcluster of the H-cluster, but are instead artifacts generated by oxidized NaDT. This result challenges their involvement in the catalytic cycle of [FeFe] hydrogenases. Furthermore, these findings highlight the importance of carefully considering the possible side-reactions of NaDT and its oxidation products when choosing to use this reducing agent with metalloenzymes, particularly iron–sulfur enzymes.

## Results

### Treatment of *Cr*HydA1 with oxidized NaDT causes formation of the HoxH state

Our investigation on the effect of the oxidation products of NaDT on [FeFe] hydrogenases focused, in the first instance, on *Cr*HydA1, the most well characterized [FeFe] hydrogenase, which contains only the H-cluster. In particular, the enzyme containing the native [2Fe] cofactor with the ADT ligand (*Cr*HydA1^ADT^) was used. Thus, *Cr*HydA1^ADT^ produced in the strict absence of NaDT was treated with a solution of oxidized NaDT (oxNaDT). This solution was prepared by dissolving fresh NaDT in water to a concentration of 1 M (the most effective concentration of NaDT for H_ox_H formation at pH 6^27^) under aerobic conditions and stirring for 2 h under atmospheric oxygen. A decrease in the pH to ∼2 and appearance of a yellow precipitate (most likely elemental sulfur) indicated oxidation and degradation of the dithionite anion.^47^ The oxNaDT solution was then thoroughly degassed and moved into an anaerobic glovebox before being added to *Cr*HydA1^ADT^, in order to avoid damaging the highly air-sensitive H-cluster.

**Figure 2:**
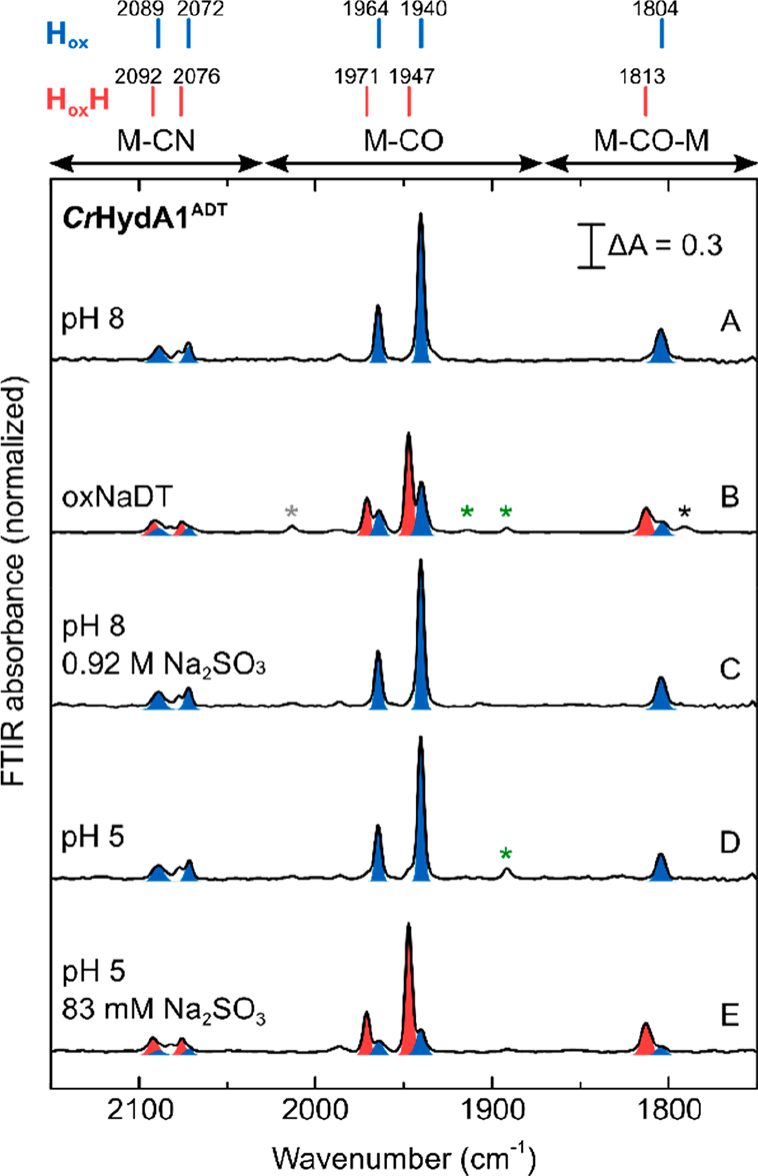
IR spectra of *Cr*HydA1^ADT^ showing formation of H_ox_H with oxNaDT and Na_2_SO_3_ at low pH. IR spectra of *Cr*HydA1^ADT^ were measured under a N_2_ atmosphere at room temperature in 20 mM mixed buffer (see experimental section) pH 8 (A), in 0.83 M acidic oxNaDT (B), 0.92 M Na_2_SO_3_ pH 8 (C), in 20 mM mixed buffer pH 5 in the absence (D), or the presence (E) of 83 mM Na_2_SO_3_. Spectra were normalized to allow easier comparison from different measurements. Peaks for the H_ox_ and H_ox_H states are highlighted in blue and red, respectively. Small contributions from the H_ox_-CO (gray asterisk), H_red_H^+^ (green), and H_red_ (black) states are indicated. Importantly, Na_2_SO_3_ solutions were pH corrected before use, whereas the solution of oxNaDT was not pH corrected, but measured to be around 2.

As shown by the IR spectra of *Cr*HydA1^ADT^ (Figure 2), dilution of the enzyme in the oxNaDT solution results in the appearance of a new set of vibrational signals, slightly shifted to higher energy (<10 cm^–1^) with respect to H_ox_. These new signals are consistent with those reported for H_ox_H.^10,19,27^ Even though the pH of the oxNaDT solution was measured to be around 2, the buffer present in the *Cr*HydA1^ADT^ sample (25 mM Tris-HCl, pH 8) will render the pH value after oxNaDT addition slightly higher than this (ca. pH 6). Interestingly, when *Cr*HydA1^ADT^ was treated with a solution oxNaDT whose pH had been corrected to 7, conversion to H_ox_H was not observed (Figure S1), suggesting that formation of this state requires acidic conditions.

The observation that H_ox_H can be formed by treatment with oxidized NaDT challenges the hypothesis that H_ox_H and H_red_′H are protonated versions of the H-cluster. However, it was not clear which component of oxNaDT was interacting with the H-cluster. To better understand the nature of these two states and their role in the catalytic cycle of [FeFe] hydrogenases, we sought to identify the oxidation product(s) of NaDT responsible for their formation.

### H_ox_H forms in the presence of sulfite at low pH

The main oxidation and degradation products of NaDT are sulfate (SO_4_^2–^), thiosulfate (S_2_O_3_^2–^), and sulfite (SO_3_^2–^),^48,49^ all of which could potentially interact with the H-cluster of *Cr*HydA1 and cause conversion to the H_ox_H state. Therefore, to identify the NaDT oxidation products responsible for this conversion, we tested these species individually on *Cr*HydA1 at both pH 8 and pH 5. Treatment of *Cr*HydA1 with Na_2_SO_4_ and Na_2_S_2_O_3_ at either pH 8 or pH 5 failed to reproduce the H_ox_H state (Figure S2). In contrast, we found that addition of 80 mM of Na_2_SO_3_ at pH 5 reproduced the effect of oxNaDT and caused almost full conversion to the H_ox_H state, while at pH 8 even a high concentration (0.92 M) of Na_2_SO_3_ had no effect on *Cr*HydA1 (Figure 2). Importantly, *Cr*HydA1 at pH 5 before addition of Na_2_SO_3_ has an identical spectrum to that at pH 8, demonstrating that both low pH and Na_2_SO_3_ are required for H_ox_H formation. Na_2_SO_4_, Na_2_S_2_O_3_, and Na_2_SO_3_ solutions were pH corrected before use—this is particularly important for Na_2_SO_3_, which is a mild base.

In addition to *Cr*HydA1, also the bacterial [FeFe] hydrogenases HydAB from *Desulfovibrio desulfuricans* (*Dd*HydAB) and HydA1 from *Clostridium pasteurianum* (*Cp*HydA1) have been reported to form the H_ox_H state at low pH and in the presence of NaDT.^27^ These enzymes harbor additional [4Fe-4S] clusters (F-clusters) that form an electron-transfer chain from the protein surface to the H-cluster, and compared to *Cr*HydA1, their active site is deeply buried inside the protein scaffold.^3,4^ When treated with Na_2_SO_3_ under acidic conditions, also *Dd*HydAB and *Cp*HydA1 converted to the H_ox_H state (Figure S3), indicating that the interaction of the H-cluster with the oxidation product of NaDT is a generalized phenomenon in [FeFe] hydrogenases.

### A protonated form of sulfite interacts with the H-cluster

**Figure 3:**
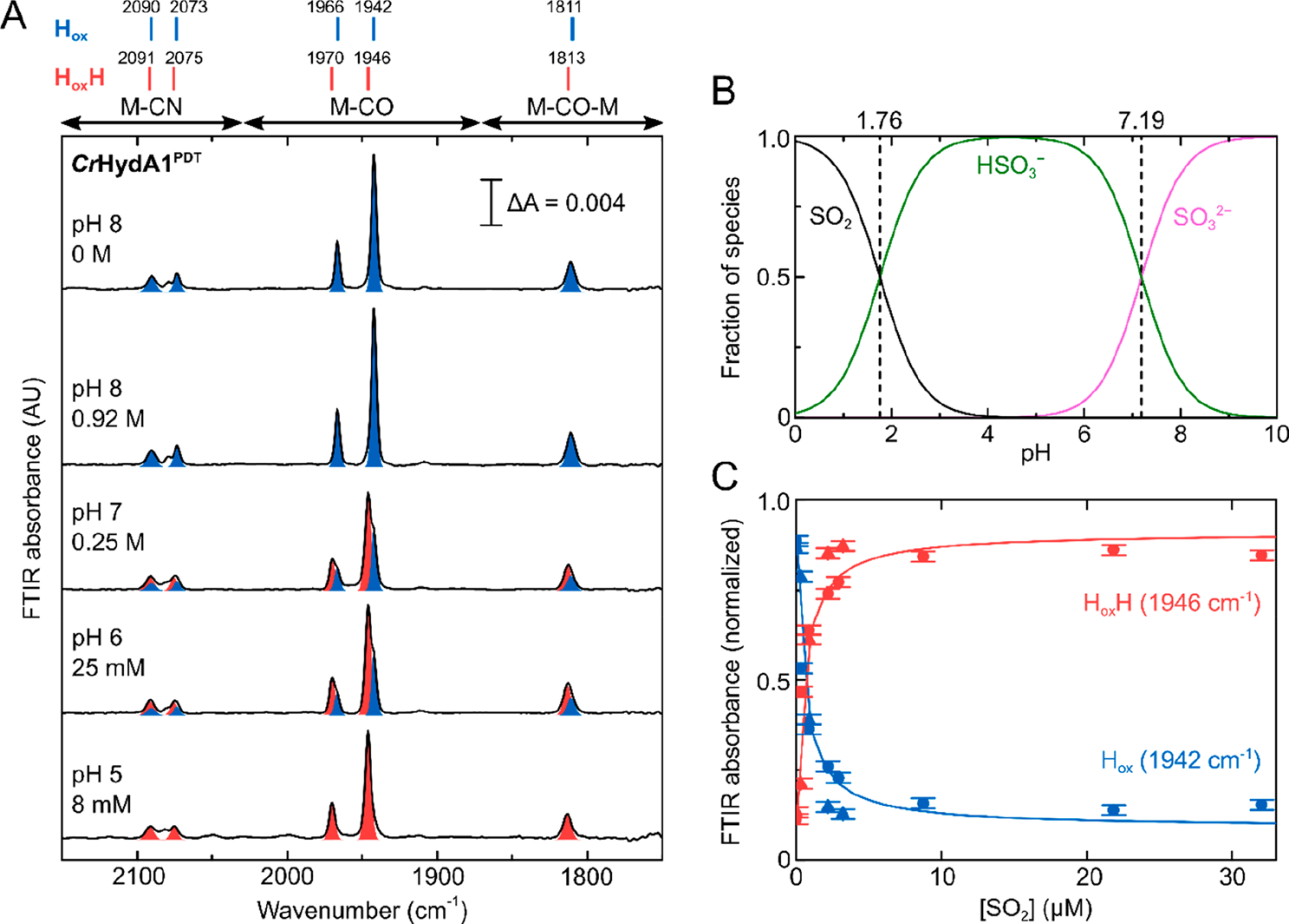
Titration of *Cr*HydA1^PDT^ with Na_2_SO_3_ under N_2_ at various pH values. (A) IR spectra are shown for a range of conditions (pH 5–8) under various concentrations of Na_2_SO_3_ (0–0.92 M). The peaks for the H_ox_ and H_ox_H states are highlighted in blue and red, respectively. (B) Predicted speciation of sulfite in water as a function of the pH assuming an acid dissociation constant (p*K*_a_) of 7.19 for HSO_3_^–^ ⇌ H^+^ + SO_3_^2–^ and an equilibrium constant (p*K*) of 1.76 for SO_2_ + H_2_O ⇌ H^+^ + HSO_3_^–^.^50^ (C) Variation in the intensity of the 1942 cm^–1^ (H_ox_) and 1946 cm^–1^ (H_ox_H) peaks with the estimated concentration of dissolved SO_2_ at pH 7 (triangles) and 6 (circles). The data were fitted with a model describing binding of SO_2_ to the hydrogenase with 1:1 stoichiometry and assuming that the concentration of SO_2_ at equilibrium is determined only by the pH and the concentration of Na_2_SO_3_. The data at pH 6 and 7 were fitted simultaneously to the same model. For an expanded version of the region from 0 to 6 μM SO_2_; see Figure S6E. Error bars (±standard deviation) were determined by measuring the 0, 0.25, and 0.92 M Na_2_SO_3_ spectra at pH 7 and the 25 mM Na_2_SO_3_ spectrum at pH 6 in triplicate, which gave standard deviations of less than 0.014.

Next, we decided to carry out titrations of *Cr*HydA1 with Na_2_SO_3_ at various pH values in order to provide further details on the particular form of Na_2_SO_3_ that binds, as well as determining the binding affinity. In order to simplify the titrations, we chose to use a chemical variant of *Cr*HydA1 with a [2Fe]_H_ analogue containing a propane dithiolate (PDT) bridging ligand instead of ADT (*Cr*HydA1^PDT^, Figure 1A). Compared to the amine in ADT, the methylene group in PDT cannot be easily protonated. As a result, *Cr*HydA1^PDT^ has very low catalytic activity and the H-cluster cannot assume states with a reduced [2Fe]_H_ (i.e., H_red_H^+^ and H_sred_H^+^) (Figure 1C). This greatly reduces the number of states observable in the IR spectra, simplifying data analysis. The PDT-containing enzyme was previously shown to convert to H_ox_H and H_red_′H at low pH in the presence of NaDT.^10,27^*Cr*HydA1^PDT^ was titrated with increasing amounts of Na_2_SO_3_ at five different pH values (Figure 3 and Figures S4–S6). In an anaerobic glovebox with a 100% N_2_ atmosphere, the H-cluster was in the oxidized state H_ox_ at the beginning of the titration for all the pH values tested. As already observed for native *Cr*HydA1^ADT^, at pH 8 addition of even a very high concentration of Na_2_SO_3_ did not affect the state of the H-cluster, which remained in the H_ox_ state. Conversely, at pH 7, H_ox_H appeared already with less than 250 mM Na_2_SO_3_, and complete conversion was observed at around 700 mM. The concentration of Na_2_SO_3_ needed in order to observe complete conversion from H_ox_ to H_ox_H decreased at pH 6 to about 200 mM and at pH 5 to less than 8 mM. At pH 4, 1 mM Na_2_SO_3_ gave essentially complete conversion to H_ox_H, while 1 mM Na_2_SO_3_ at pH 5 gave a roughly equal mixture of H_ox_ and H_ox_H (Figure S5).

In aqueous solutions SO_3_^2–^ is in equilibrium with its protonated form bisulfite (HSO_3_^–^), which in turn can be further protonated to form sulfurous acid (H_2_SO_3_), which immediately decomposes to sulfur dioxide (SO_2_) and water (Figure 3B).^51−53^ As Figure 3 shows, the lower the pH, the lower the concentration of sulfite needed to convert H_ox_ to H_ox_H. This, therefore, excludes that SO_3_^2–^, whose abundance is predicted to greatly decrease when changing the pH from 8 to 6, is responsible for formation of H_ox_H. Since, as shown in Figure 3A, lowering the pH from 6 to 5, and then to 4 (Figure S5), caused a further reduction in the required concentration of Na_2_SO_3_ needed to convert H_ox_ to H_ox_H, while the fraction of HSO_3_^–^ should be constant in this range (Figure 3B), HSO_3_^–^ is also unlikely to be the form of Na_2_SO_3_ binding to the H-cluster. In a pH titration of Na_2_SO_3_ monitored by IR spectroscopy we observed that the intensity of peaks relative to HSO_3_^–^ indeed saturated after pH 6.0–5.5, while signals indicative of the presence of SO_2_ appeared at pH 5 (Figure S7). Therefore, we hypothesize that the species interacting with the H-cluster to form H_ox_H is SO_2_. This seems reasonable considering that SO_2_ is a neutral molecule able to easily diffuse through hydrophobic channels^54,55^ to reach the H-cluster from the protein surface, while the anions HSO_3_^–^ and SO_3_^2–^ will be prevented from entering due to their charge and their large hydration spheres in aqueous solution.^56^ A similar suggestion was made to explain how S^2–^ reaches the H-cluster as H_2_S to form the H_inact_ state.^57^

At pH 7 and 6, even at high concentration of sulfite, the concentration of dissolved SO_2_ is expected to be very low. Thus, in order to observe binding to the H-cluster and formation of H_ox_H, SO_2_ must have a tight affinity for the enzyme. Figure 3C shows the conversion from H_ox_ to H_ox_H as a function of the estimated concentration of SO_2_ at each Na_2_SO_3_ addition, at either pH 6 or 7. The population of the two states was monitored from the intensity of the most prominent CO band at 1942 cm^–1^ for H_ox_ and 1946 cm^–1^ for H_ox_H, in both cases corresponding to the stretch of the terminal CO on Fe_d_. The titration curves at pH 7 and 6 as a function of the concentration of SO_2_ overlay nicely, in contrast to those obtained using the estimated concentrations of HSO_3_^–^ and SO_3_^2–^ (Figure S6). Fitting the data in Figure 3C to a simple equilibrium model describing one SO_2_ molecule binding to the hydrogenase (SO_2_ + E ⇌ E:SO_2_) gave an estimated binding affinity of ∼500 nM. In our analysis, we considered that the pool of Na_2_SO_3_ can act as a buffer system for SO_2_, replenishing what is consumed to form the enzyme:SO_2_ complex (E:SO_2_). For all the data points, the concentration of E:SO_2_ formed was negligible compared to the total concentration of Na_2_SO_3_, so that the concentration of SO_2_ at equilibrium could be assumed to be independent of the formation of E:SO_2_ and to be determined only by the pH and the total concentration of Na_2_SO_3_, an important consideration for such tight binding interactions. To put this in context, CO has been estimated to bind with 100 nM affinity to *Cr*HydA1^ADT^.^58^

### Addition of sulfite under reducing conditions (H_2_ atmosphere) forms H_red_′H

**Figure 4:**
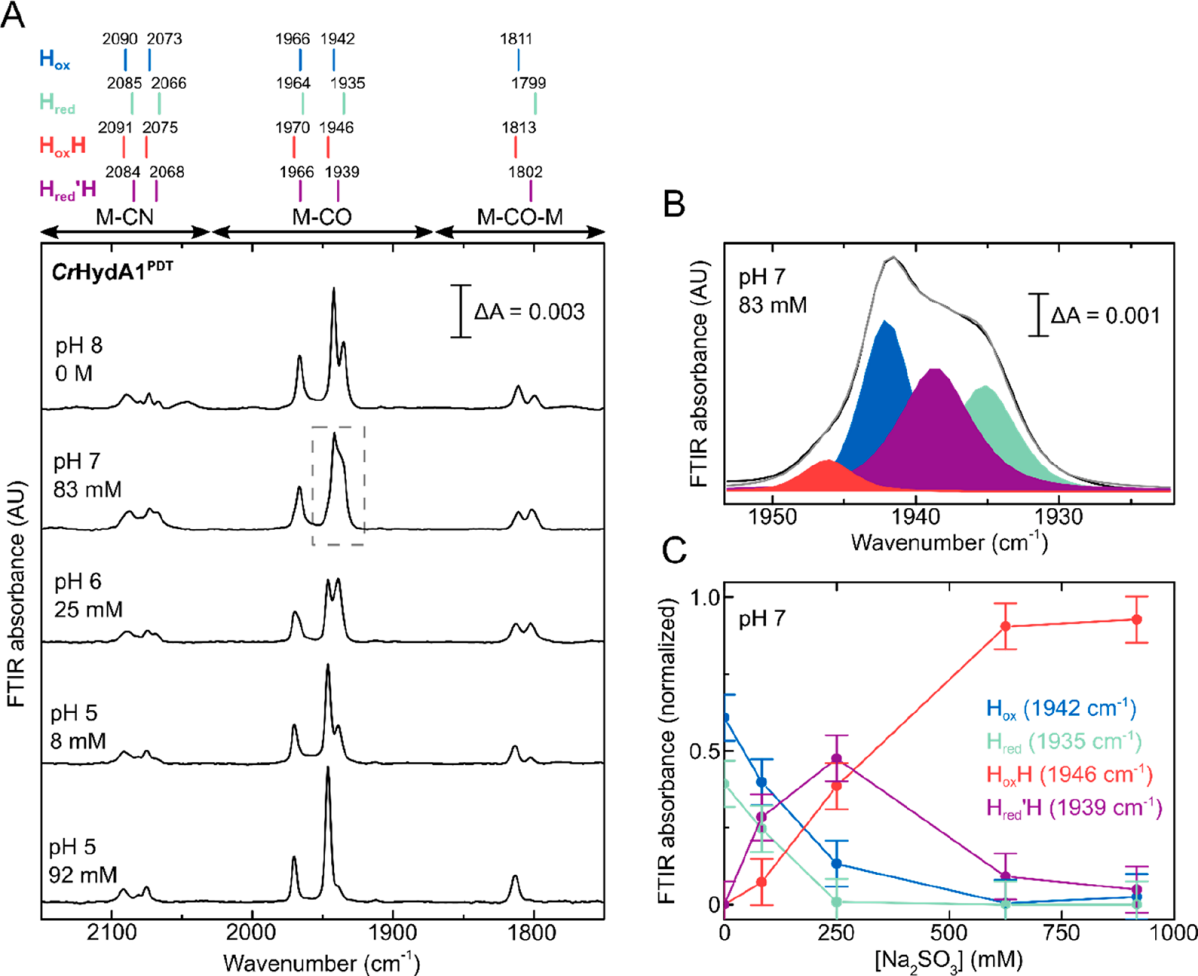
Titration of *Cr*HydA1^PDT^ with Na_2_SO_3_ at various pH values under 2% H_2_. (A) IR spectra are shown for a range of conditions (pH 5–8) under various concentrations of Na_2_SO_3_ (0–0.92 M). (B) Peak-fitting to pseudo-Voigt functions of the region between 1955 and 1920 cm^–1^ for the data in the dashed rectangle in A. Color code: H_ox_ blue, H_red_ cyan, H_ox_H red, H_red_′H purple. (C) The variation in the intensity of the 1942 cm^–1^ (H_ox_), 1935 cm^–1^ (H_red_), 1946 cm^–1^ (H_ox_H), and 1939 cm^–1^ (H_red_′H) peaks with the Na_2_SO_3_ concentration at pH 7. The lines connecting the points in C are for visual purposes only. Error bars (±standard deviation) were determined by measuring the 0, 0.25, and 0.92 M Na_2_SO_3_ spectra at pH 7 in triplicate, which gave standard deviations of less than 0.075.

The titration of *Cr*HydA1^PDT^ with sulfite was repeated in the presence of 2% H_2_ in the atmosphere of the anaerobic glovebox (Figure 4). Under these conditions, slow reactivity of the *Cr*HydA1^PDT^ enzyme with H_2_ can lead to reduction of the [4Fe-4S]_H_ subcluster, in particular at high pH values. This is due to the potential of the 2H^+^/H_2_ couple, which becomes more positive as the pH decreases, while the redox potential of [4Fe-4S]_H_ is pH independent.^12^ At pH 7, after addition of a small amount of Na_2_SO_3_, we observed a mixture of the H_ox_, H_red_, and H_ox_H states in the IR spectra, plus a new set of signals. These are consistent with the vibrational frequencies of the H_red_′H state, which Stripp and co-workers reported to form with NaDT at low pH and either under H_2_ or at low electrochemical potential.^10,27^ Similar to what was observed under N_2_, at lower pH the formation of H_ox_H and H_red_′H was observed at lower concentration of Na_2_SO_3_. In order to estimate the proportion of each state present under each condition, we performed a pseudo-Voigt peak-fitting analysis of the region of the spectrum between ∼1955 cm^–1^ and ∼1920 cm^–1^, containing the most dominant bands for H_ox_ (1942 cm^–1^, blue), H_red_ (1935 cm^–1^, cyan), H_ox_H (1946 cm^–1^, red), and H_red_′H (1939 cm^–1^, purple) (Figures 4B, S8–S10). In Figure 4C, the intensity of these contributions is plotted as a function of the concentration of Na_2_SO_3_ at pH 7. At low Na_2_SO_3_, both the H_ox_H and H_red_′H states are observed, but at high concentrations of Na_2_SO_3_ H_red_′H is converted to H_ox_H. This indicates oxidation of the [4Fe-4S]_H_ subcluster by Na_2_SO_3_. Since the samples were prepared in a closed IR cell and the concentrations of sulfite used are much higher than the dissolved concentration of H_2_, oxidation by Na_2_SO_3_ will slowly deplete the H_2_ concentration leading to oxidation of the sample. Similar behavior is observed also at pH 6 and 5 (Figures S8, S10).

At low concentrations (8 mM) of Na_2_SO_3_, at pH 6, the H_red_′H is the most dominant state, while H_ox_H becomes more favored at pH 5 at the same concentration of Na_2_SO_3_, agreeing with a pH independent redox potential of [4Fe-4S]_H_ also when SO_2_ is bound. However, the fact that SO_2_ is more prevalent at low pH gives the conversion of H_ox_/H_red_ to H_ox_H/H_red_′H an “apparent” pH dependence. This will complicate the interpretation of pH-dependent redox titrations performed in the presence of oxidation products of sodium dithionite (including Na_2_SO_3_ and SO_2_), which may explain discrepancies in the literature.^10,12^

**Scheme 1:**
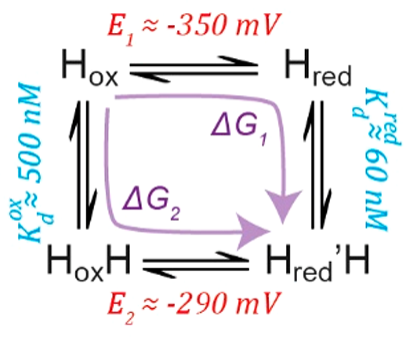
Thermodynamic Cycle Connecting H_ox_, H_red_, H_ox_H, and H_red_′H One-electron reduction of H_ox_ and H_ox_H gives H_red_ and H_red_′H, respectively, with redox potentials of *E*_1_ ≈ −350 mV and *E*_2_ ≈ −290 mV, respectively. H_ox_ and H_red_ convert to H_ox_H and H_red_′H, respectively, by binding SO_2_. The *K*_d_ for SO_2_ binding to the H_ox_ state was measured to be ∼500 nM. By consideration of the fact that the Gibbs free energy is a state function, the Δ*G* associated with the transition from H_ox_ to H_red_′H is the same regardless of whether we go via H_red_ (*ΔG*_1_) or via H_ox_H (*ΔG*_2_), allowing us to calculate the *K*_d_ for binding of SO_2_ to the H_red_ state to be ∼60 nM.

Interestingly, at low concentrations of Na_2_SO_3_, the ratio of H_ox_:H_red_ is much greater than that of H_ox_H:H_red_′H, suggesting that binding of SO_2_ increases the redox potential of the [4Fe-4S]_H_ subcluster (Figure 4B and S8, S9). The redox potential for the H_ox_/H_red_ and H_ox_H/H_red_′H transitions can be calculated at pH 6 and 7 at low concentrations of Na_2_SO_3_ from the populations of the four states (Figure S11). Using the Nernst equation, we found that *E*_m_ (H_ox_/H_red_) = −349 (±17) mV and *E*_m_ (H_ox_H/H_red_′H) = −293 (±26) mV. The value for *E*_m_ (H_ox_/H_red_) is in close agreement with that determined previously.^12,59^ The fact that the redox potential for the H_ox_H/H_red_′H transition is ∼60 mV more positive than the H_ox_/H_red_ transition also indicates a tighter binding affinity for SO_2_ to the H_red_ state than to the H_ox_ state. We determined a *K*_d_ for SO_2_ binding to the H_ox_ state of ∼500 nM from the titrations in the absence of H_2_. By considering the thermodynamic cycle (Scheme 1) connecting the H_ox_, H_red_, H_ox_H, and H_red_′H states, it can be calculated that an ∼60 mV difference in the redox potentials indicates a *K*_d_ of ∼60 nM for SO_2_ binding to the H_red_ state, approximately 1 order of magnitude tighter. This also means that low concentrations of Na_2_SO_3_ have a larger effect in the presence of H_2_ (compare Figure 4B with Figure S4B).

### The site of SO_2_ binding is not the open coordination site on [2Fe]_H_

From the previous section, it is clear that SO_2_ somehow interacts with the H-cluster of [FeFe] hydrogenases. It is tempting to speculate that SO_2_ diffuses through the hydrophobic gas channel leading to the open coordination site on Fe_d_. However, we cannot exclude that SO_2_ binds elsewhere, and indeed, the change in the redox potential of [4Fe-4S]_H_ would suggest that binding near to [4Fe-4S]_H_ is more likely. To test whether binding of SO_2_ with the H-cluster occurs at the open coordination site on [2Fe]_H_, we investigated how its presence can affect the interaction of the enzyme with CO, a competitive inhibitor of [FeFe] hydrogenases that binds to Fe_d_.^60^ At pH 5 exposure of *Cr*HydA1^ADT^ to 100% CO gas for 10 min in the absence of Na_2_SO_3_ generates pure H_ox_-CO (Figure S12). In the presence of a high concentration of sulfite at pH 5, exposure of *Cr*HydA1 to CO caused the appearance of new peaks that correspond to neither H_ox_-CO nor H_ox_H, and are similar to the H_ox_H–CO state described by Stripp and co-workers (Figure S12).^27^ This suggests that SO_2_ does not compete for the same binding site as CO, which is the open coordination site at Fe_d_.

**Figure 5:**
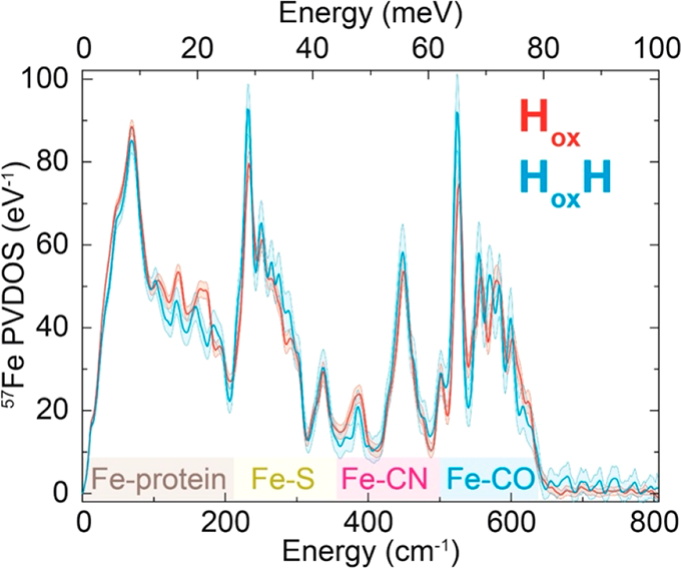
Comparison of the NRVS spectra of *Cr*HydA1 maturated with an ^57^Fe-labeled [2Fe]^ADT^ precursor complex in the H_ox_ (red) and H_ox_H (blue) states measured at 10 K. The regions of the spectra corresponding to Fe-protein, Fe–S, Fe-CN, and Fe-CO vibrations are highlighted in brown, yellow, pink, and blue along the *x*-axis.

In order to get further information on the SO_2_ binding site, we measured ^57^Fe nuclear resonance vibrational spectroscopy (NRVS). This technique measures Fe-ligand vibrational energies using nuclear excitation of ^57^Fe and has been used extensively to probe ligand binding to the [2Fe]_H_ subcluster in [FeFe] hydrogenase.^21−23,28,57^ We artificially maturated apo-*Cr*HydA1 samples with a ^57^Fe-labeled diiron subcluster precursor ([2^57^Fe]^ADT^) and measured NRVS in the H_ox_ and H_ox_H states (Figure 5). This enzyme is labeled with ^57^Fe in the [2Fe]_H_ subcluster and not in the [4Fe-4S]_H_ subcluster, so only vibrations involving motion of the [2Fe]_H_ subcluster can be observed. The spectra of H_ox_ and H_ox_H are very similar but with small shifts of the peaks to lower energy for the H_ox_H, indicative of decreased electron density on the [2Fe]_H_ subcluster, similar to results observed by Mebs *et al.*(28) In contrast, ligand binding to Fe_d_ on the [2Fe]_H_ subcluster would be expected to have much more dramatic changes, particularly, the generation of additional Fe–S or Fe–O vibrations.^57,61^ The results cannot definitively confirm the [4Fe-4S]_H_ subcluster as the point of SO_2_ binding, but together with the observation of the H_ox_H–CO state, they do exclude the open coordination site on Fe_d_ as the SO_2_ binding site.

### SO_2_ inhibits catalysis by [FeFe] hydrogenase

**Figure 6:**
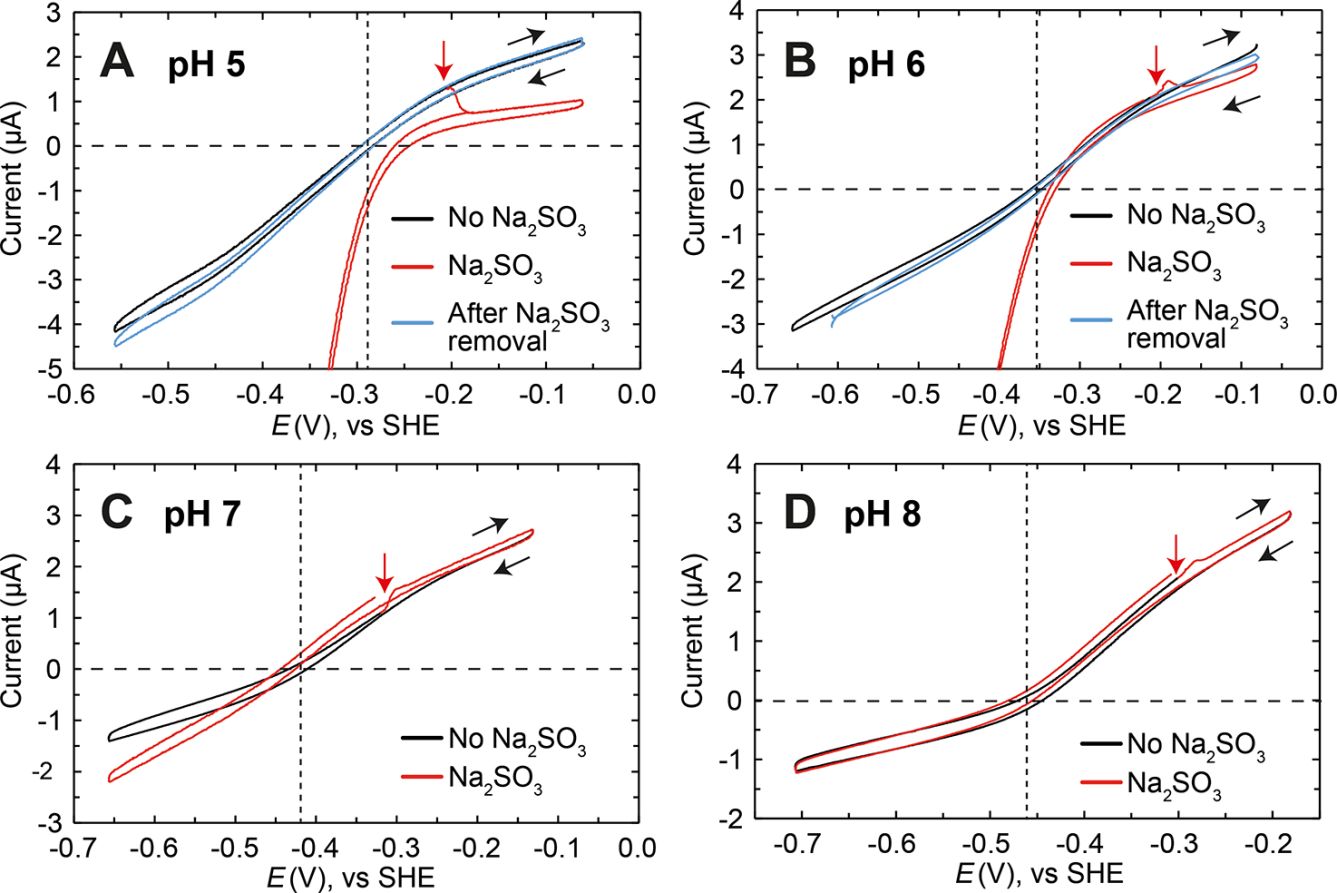
Protein film electrochemistry of *Dd*HydAB in the presence of sulfite. *Dd*HydAB was covalently attached to a pyrolytic graphite electrode and cyclic voltammetry (CV) was performed in 20 mM mixed buffer with 100 mM NaCl at pH 5 (A) 6 (B), 7 (C), and 8 (D) under 100% H_2_ (1000 mL/min), at 25 °C, with 2000 rpm rotation, and a scan rate of 0.02 V/s. After 3 CVs in the absence of Na_2_SO_3_ (only the third trace is shown, black trace), 40 mM Na_2_SO_3_ was added (red trace). Only a single CV before and after the addition of Na_2_SO_3_ are shown. However, consecutive CVs showed the same shape. After replacing the buffer in the electrochemical cell with Na_2_SO_3_-free buffer, *Dd*HydAB recovered its original activity (blue traces). The red arrow indicates the point of Na_2_SO_3_ injection. The black arrows indicate the scan direction of the CV. The dashed horizontal line shows the zero current position, and the dashed vertical line shows the equilibrium 2H^+^/H_2_ potential at each pH value. Enlarged versions of A and B are reported in Figure S13.

In order to investigate the effect of SO_2_-binding to the H-cluster on catalysis, we performed protein film electrochemistry on the *Dd*HydAB enzyme covalently attached to a pyrolytic graphite electrode. We chose *Dd*HydAB rather than *Cr*HydA1, as the former is, in our hands, much easier to covalently attach to graphite electrode surfaces.^62^ As shown in the cyclic voltammograms (CVs) in Figure 6 and in the enlarged version of the CVs reported in Figure S13, a large negative current at low potentials is observed when Na_2_SO_3_ is injected into the electrochemical cell under acidic conditions (pH 5 and pH 6, respectively A and B in Figure 6). Controls experiments (bare graphite electrode injecting Na_2_SO_3_, Figure S14) suggest that this reduction current is likely due to HSO_3_^–^ and SO_2_ being reduced by the pyrolytic graphite electrode.^63^ Comparisons of bare graphite electrodes and *Dd*HydAB-modified electrodes at various pH values are presented in the absence (Figure S15) and presence (Figure S16) of Na_2_SO_3_. Unfortunately, this massive reduction current masks the effect of Na_2_SO_3_ on the catalytic H^+^-reduction current.

However, as shown in Figure 6A and B (CV at pH 5 and 6 in the presence of Na_2_SO_3_, respectively), in the presence of Na_2_SO_3_ the catalytic H_2_-oxidation current decreases, suggesting inhibition of the enzyme as a result of the H-cluster somehow interacting with SO_2_. The inhibitory effect on the catalytic H_2_-oxidation current is more pronounced at lower pH (the CVs at pH 7 and 8 are reported in Figure 6C and D, respectively), in agreement with the pH-dependent formation of H_ox_H and H_red_′H observed in the IR measurements. To explore whether the inhibition is reversible and the electrocatalytic H_2_-oxidation current can be recovered, the buffer in the electrochemical cell was exchanged to a fresh buffer without Na_2_SO_3_ during the course of the CVs. Sulfite-exposed *Dd*HydAB recovered 100% of the electrocatalytic H_2_-oxidation current once Na_2_SO_3_ was removed from the electrochemical cell, suggesting that SO_2_ binding and inhibition are fully reversible (blue trace in Figure 6A and B) and that the enzyme is not irreversibly damaged by SO_2_.

**Figure 7:**
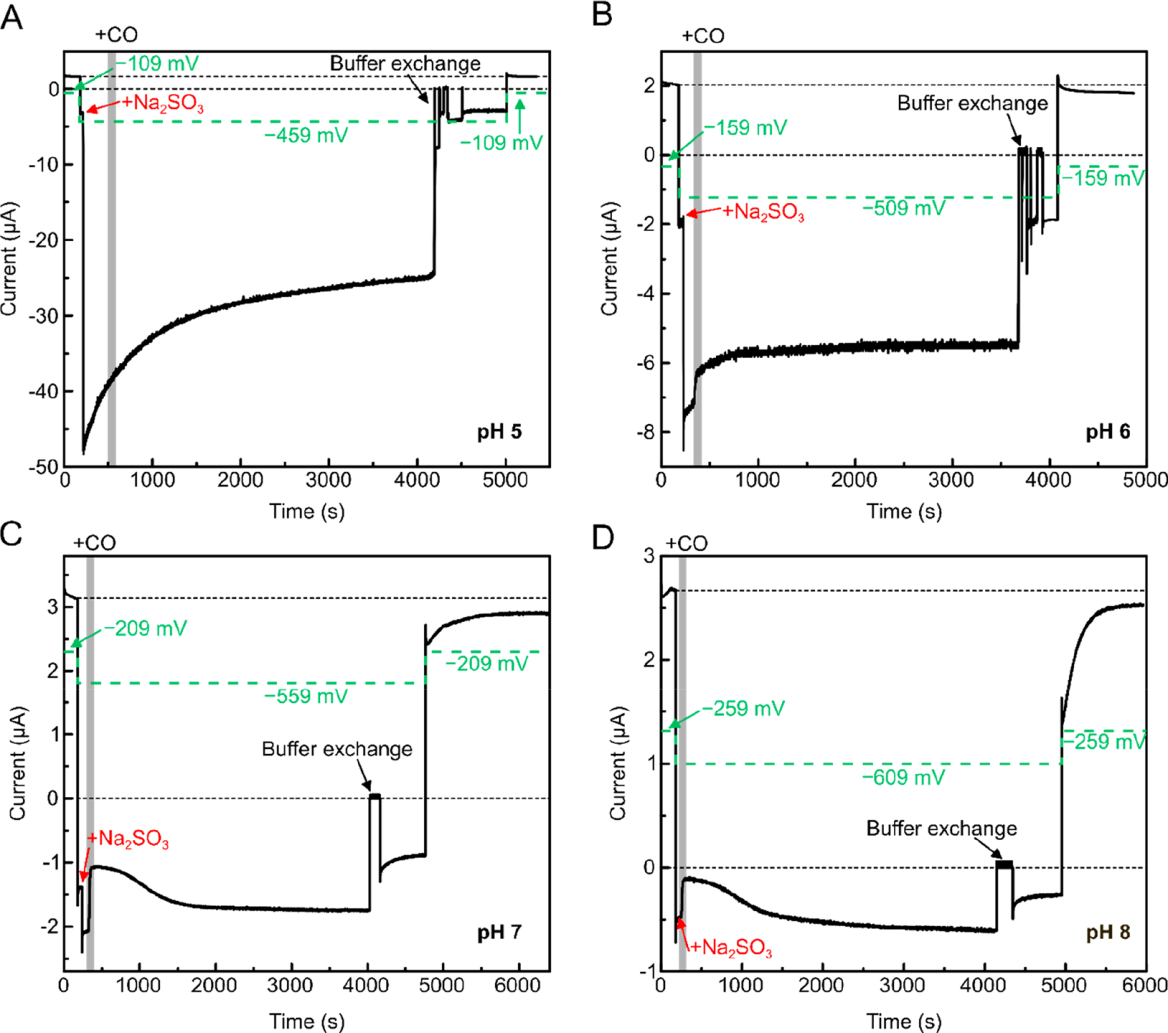
Chronoamperometry experiments of *Dd*HydAB in the presence of Na_2_SO_3_ and CO. Chronoamperometry experiments were performed on *Dd*HydAB covalently attached to a pyrolytic graphite electrode under 1 bar 90% H_2_ in N_2_ (1000 mL/min), in 20 mM mixed buffer with 100 mM NaCl at pH 5 (A), pH 6 (B), pH 7 (C), pH 8 (D) at 25 °C and 2000 rpm rotation rate. During the experiment the potential was sequentially stepped as indicated by the green profile (all potentials are reported *vs* SHE). For example, at pH 5 (A) the potential was initially set to −109 mV, next stepped down to −459 mV, and finally back to the initial potential −109 mV. Addition of 40 mM Na_2_SO_3_ is indicated by red arrows, while addition of 10% CO (in 90% H_2_) to the gas mixture is indicated by the shaded gray area. After more than 3600 s, the buffer inside the electrochemical cell was rinsed and exchanged with fresh buffer without Na_2_SO_3_. Note the complex behavior in the region immediately following CO treatment at pH 6 in (B). This represents a convolution of the current recovery due to CO release and the exponential decay of the current as a result of decreasing Na_2_SO_3_ reduction. To observe the current recovery due to CO release, a simulated exponential decay curve was subtracted from the experimental data (Figure S17C), and the resulting difference curve is plotted in Figure S17D.

The massive current at low potential due to direct reduction of HSO_3_^–^ and SO_2_ species by the electrode makes it difficult to assess the effect of Na_2_SO_3_ on the electrocatalytic H^+^-reduction current. To distinguish the enzymatic contribution from the direct HSO_3_^–^ and SO_2_ reduction by the electrode, we performed chronoamperometry experiments (the applied potential is held at a specific value while the current is monitored *vs* time) in the presence and absence of CO (Figure 7). As previously described,^58,62^ the current decrease due to CO addition (as CO binds to open coordination site on Fe_d_ and inhibits the enzyme) provides a direct measurement of the enzymatic H^+^ reduction. In the experiment in Figure 7A, performed at pH 5, *Dd*HydAB attached on the pyrolytic graphite electrode was initially exposed to 90% H_2_/10% N_2_ at −109 mV *vs* SHE, where a positive current due to H_2_ oxidation was observed (as the applied potential is more positive than the thermodynamic potential of the 2H^+/^H_2_ couple at this pH, −295 mV *vs* SHE). Switching to −459 mV gave a small negative current due to H^+^ reduction (as the applied potential is now more negative than *E*_2H^+^/H_2__ at this pH). Adding Na_2_SO_3_ at this potential gave an extremely large negative current, which was unaffected by addition of 10% CO into the gas feed (replacing the 10% N_2_). This indicates that the large negative current is entirely due to Na_2_SO_3_ reduction and that catalytic H^+^ reduction by *Dd*HydAB is completely inhibited under these conditions. Replacing the buffer with fresh Na_2_SO_3_-free buffer decreased the current to the original value observed before addition of Na_2_SO_3_. An analogous experiment at pH 6 (Figure 7B) showed a small decrease in the current after addition of CO, as well as experiments at pH 7 and pH 8 (Figure 7C and 7D, respectively), suggesting that at these pH values there is some contribution from the enzymatic H^+^ reduction current, in agreement with the pH dependent formation of SO_2_ from Na_2_SO_3_. Control experiments in the complete absence of Na_2_SO_3_ showed full inhibition of the electrocatalytic H^+^-reduction current by CO, thus demonstrating that in the absence of Na_2_SO_3_ the reductive current is indeed enzymatic H^+^ reduction (Figure S17). At this stage, it is unclear whether the loss in activity in both directions due to Na_2_SO_3_ addition is directly related to the increase in the redox potential of [4Fe-4S]_H_. The higher redox potential of the cluster may disrupt the proton-coupled electronic rearrangement between [4Fe-4S]_H_ and [2Fe]_H_.^14^ These experiments help to understand the discrepancy between reported H^+^ reduction activity solution assays and electrochemistry. While solution assays (where NaDT is used as electron source) indicate a maximum in activity at pH 7,^8^ and almost no activity at pH 5, electrochemical measurements show the highest H^+^ reduction activity at pH 5 (Figure S15). Regardless, these data show that, under the conditions where H_ox_H and H_red_′H form, the enzyme has lower activity, suggesting that these states are not active intermediates of the catalytic cycle of [FeFe] hydrogenases. This is in stark contrast to the suggestion from Stripp and Haumann that a catalytic cycle involving H_ox_H is actually the faster branch of the cycle compared to that involving the H_red_H^+^ and H_sred_H^+^ states (Figure 1D).

## Discussion

In this work we have shown that in *Cr*HydA1 the H_ox_H state forms in the presence of oxidation products of NaDT at low pH, specifically SO_2_. SO_2_ binding caused formation of H_ox_H not only with *Cr*HydA1 but also with the bacterial enzymes *Cp*HydA1 and *Dd*HydAB, suggesting this is a common behavior in [FeFe] hydrogenases. Additionally, we have shown that with Na_2_SO_3_ and in the presence of H_2_ the reduced H_red_′H state can also form. The electrochemistry measurements showed loss in electrocatalytic activity when *Dd*HydAB was exposed to Na_2_SO_3_, especially at low pH, suggesting that H_ox_H and H_red_′H are less active states and challenging their inclusion in the catalytic cycle. Taken together, these findings suggest that H_ox_H and H_red_′H are not protonated versions of H_ox_ and H_red_, but instead are forms of H_ox_ and H_red_ in which a product of NaDT oxidation, most likely SO_2_, is bound. Thus, we suggest renaming H_ox_-DT_i_ and H_red_-DT_I_ (for dithionite inhibited) to avoid confusion, and for the rest of the discussion we will name them as such.

This result helps explain previous findings in the literature regarding these states. Originally, H_ox_-DT_i_ and H_red_-DT_i_ were discovered during NaDT-mediated H^+^ reduction by [FeFe] hydrogenase at low pH.^19,27^ Under these conditions H^+^ reduction rates are high, leading to rapid oxidation of NaDT to generate a mixture of SO_3_^2–^, HSO_3_^–^, and SO_2_. At low pH, SO_2_ forms due to the protonation equilibria and it can bind to the hydrogenase yielding the H_ox_-DT_i_ and H_red_-DT_i_ states. It was noticed that the accumulation of H_ox_-DT_i_ was dependent both on pH and on NaDT concentration, both of which will affect the rate of SO_2_ accumulation. Furthermore, it was noted that less active forms of the hydrogenase (e.g., with the PDT cofactor) accumulated H_ox_-DT_i_ more slowly. In this case, the accumulation of SO_2_ depends on the rate of NaDT oxidation by the catalytic activity of the hydrogenase, and it is well established that the PDT-form of the hydrogenase is catalytically much less active than the native ADT-form.^64^

Protonation at [4Fe-4S]_H_ is a critical component in the catalytic cycle proposed in Model 2 (Figure 1D). We recently demonstrated that (in the absence of NaDT) the redox potential of [4Fe-4S]_H_ is pH-independent, challenging the involvement of PCET in the formation of H_red_ and the protonation at [4Fe-4S]_H_.^12^ Our current work further challenges protonation at [4Fe-4S]_H_ by showing that the Model 2 key intermediate H_ox_-DT_i_ (H_ox_H in Figure 1D) is generated by the oxidation products of NaDT. If reduction of [4Fe-4S]_H_ is coupled to protonation then it has to be coupled to protonation in all the steps involving reduction of [4Fe-4S]_H_. Considering that the hydrogenase enzyme is reversible, with a very low overpotential in either direction, it must be assumed that each step in the catalytic cycle is also reversible and, thus, H_ox_ should be able to protonate to give H_ox_-DT_i_. However, incubation of H_ox_ at low pH in the absence of NaDT does not generate H_ox_-DT_i_ (Figure 2), so H_ox_-DT_i_ is clearly not a reversibly protonated form of H_ox_.

Our results also help to explain the misassignment of the pH dependence of the H_ox_/H_red_ transition. It is important to recall that in this study we also observe that the H_ox_-DT_i_/H_red_-DT_i_ transition is about 60 mV more positive than the H_ox_/H_red_ transition, as also reported by Senger et al.^10^ If the conversion of H_ox_ to H_ox_-DT_i_ and H_red_ to H_red_-DT_i_ depend on the pH, then we expect that the “apparent” redox potential of both transitions will shift from the intrinsic redox potential of H_ox_/H_red_ to the intrinsic redox potential of H_ox_-DT_i_/H_red_-DT_i_ as the pH is decreased. This is simply a consequence of the redox and protonation equilibria being coupled (see Supporting Information and Figure S18 for further details and a model illustrating this behavior). As we demonstrated that the SO_2_ concentration in solution increases with decreasing pH and that SO_2_ is responsible for binding to H_ox_/H_red_ to generate H_ox_-DT_i_/H_red_-DT_i_, then this gives us a pH dependent conversion of H_ox_/H_red_ to H_ox_-DT_i_/H_red_-DT_i_ and, therefore, an apparent pH dependence of the redox potential.

A further important finding regarding the [FeFe] hydrogenase is the fact that SO_2_ appears to inhibit the H_2_ oxidation and H^+^ reduction activity of the enzyme. This may be due to the increased redox potential of [4Fe-4S]_H_. While we do not yet completely understand this effect, it highlights the importance of the balance of redox potentials between the two parts of the H-cluster in facilitating electronic coupling and efficient catalysis. We previously showed that mutation of a cysteine ligating [4Fe-4S]_H_ to histidine increased the redox potential by ∼200 mV. This completely abolished H^+^ reduction activity, while actually enhancing H_2_ oxidation at high overpotentials.^65^

The now well-characterized H_hyd_ intermediate can be generated under conditions of high NaDT at low pH. It is not clear yet whether this state is also somehow influenced by the presence of SO_2_. However, it has always been intriguing how such an intermediate could be so stable by simply generating it at low pH in the presence of NaDT. Previous explanations have employed Le Chatelier’s principle and the concept of proton pressure.^19^ It may indeed be the case that SO_2_ binding stabilizes the H_hyd_ state by increasing the redox potential of the [4Fe-4S]_H_ subcluster slowing electron transfer to [2Fe]_H_ to generate H_2_. Recent evidence shows that versions of H_hyd_ can be generated from H_red_H^+^ and H_sred_H^+^.^25^ The so-called H_hyd:red_ state is generated from H_sred_H^+^ and should have a reduced [4Fe-4S]_H_ subcluster analogous to H_hyd_. Interestingly, the IR bands of H_hyd_ are shifted to higher energy compared with H_hyd:red_ by a similar amount to H_ox_-DT_i_*vs* H_ox_ (Table S2). Careful revaluation of the H_hyd_ state generated with NaDT at low pH is clearly necessary.

In addition to shedding light on the catalytic cycle of [FeFe] hydrogenases, this work reports how NaDT, a compound commonly employed as a reducing agent in metalloenzyme research, is responsible for the generation of artifacts, which were erroneously characterized as catalytically relevant states. To our knowledge, this is the first report of such “non-innocent” behavior of NaDT with [FeFe] hydrogenases, in this case caused by the interaction of one of the NaDT oxidation products with the enzyme. The experimental conditions should, thus, be carefully evaluated when NaDT is chosen as the reducing agent with these enzymes. As we have shown, acidic conditions facilitate formation of H_ox_-DT_i_, but at a high concentration of sulfite this state also forms at pH 7. Therefore, particular care must be taken when [FeFe] hydrogenase samples that contain (or contained) NaDT are studied at low pH, or in those cases where NaDT is used as a continuous source of electrons. While this is the first time that NaDT has been shown to interfere with spectroscopic studies of [FeFe] hydrogenases, several previous studies of various other metalloenzymes have reported similar effects. This problematic behavior has been attributed to several factors, from the slow kinetics of NaDT dissociation limiting the catalytic behavior to the unwanted interaction of its oxidation products with the system under study, as we described for [FeFe] hydrogenases. Importantly, the enzymes affected catalyze various reactions and harbor various metallocofactors, suggesting that it is difficult to predict which enzymes will be affected. As such, it is possible that similar effects are still going undetected for other systems. Therefore, the chemistry of NaDT and of its oxidation products should be carefully considered when choosing this compound as a reducing agent for metalloproteins research, and important control experiments should be routinely employed to identify possible side-reactions that can engage with the system under study. In the future it will also be important to evaluate alternative artificial reductants such as Ti(III) citrate (*E*^0′^ < −800 mV *vs* SHE^66^) and Eu(II)-DPTA (*E*^0′^ < −1 V *vs* SHE^67^) or the physiological redox partners for their use in hydrogenase research as well as with other metalloproteins.

Inhibition of [FeFe] hydrogenases by sulfite may not simply be an artifact but could represent a physiological mechanism for diverting electrons away from H^+^ reduction by hydrogenase and toward sulfite reduction by dissimilatory and assimilatory sulfite reductases. Here, we showed that the [FeFe] hydrogenases from *C. reinhardtii*, *D. desulfuricans*, and *C. pasteurianum* all form the H_ox_H state in the presence of sulfite, each of which contains a sulfite reductase. In *C. reinhardtii* and *C. pasteurianum*, both [FeFe] hydrogenase and sulfite reductase receive electrons from ferredoxin.^68−70^ Inhibition of the hydrogenase by sulfite would divert electrons from H^+^ reduction to sulfite reduction. In *D. desulfuricans*, [FeFe] hydrogenase supplies electrons for sulfite reduction via a membrane bound electron transport chain,^71^ and so inhibition of H_2_ oxidation could stop electron transfer to the sulfite reductase, increasing sulfite concentrations even further. However, under conditions of high H_2_S, the sulfite reductase is reversed, producing sulfite from H_2_S, leading to reverse electron transport and H^+^ reduction by [FeFe] hydrogenase. Inhibition of H^+^ reduction by sulfite would prevent this H_2_S oxidation and stop sulfite accumulation.

## Conclusions

In this work we have shown that SO_2_, an oxidation product of the commonly used nonphysiological reductant sodium dithionite, binds tightly to [FeFe] hydrogenase converting the catalytic intermediate states H_ox_ and H_red_ into the H_ox_-DT_i_ and H_red_-DT_i_ states (previously named H_ox_H and H_red_H). Thus, our results do not support the notion of protonation of the [4Fe-4S]_H_ subcluster of the H-cluster, nor that the H_ox_-DT_i_ state is a critical intermediate in the catalytic cycle. SO_2_ most likely binds at or near the [4Fe-4S] subcluster and appears to increase the cluster redox potential. This in turn may explain the observed decrease in catalytic activity. Overall, these results highlight the importance of finely tuned redox potentials for catalytic activity and reversibility. More generally, these results should come as a cautionary note to all who use sodium dithionite in metalloprotein studies without concern for its “non-innocent” effects. Sodium dithionite is routinely used in studies on a wide range of metalloenzymes including nitrogenase, CO dehydrogenase, formate dehydrogenase, and many more. Careful evaluation of results from a range of nonphysiological reductants should help to establish the effects that are artifacts from those that represent the physiological behavior of the enzyme of interest.

## Supporting Information Available

Experimental section, supplementary figures and tables, supplementary discussion (PDF)
