# Supplementary material for: The Nonphysiological Reductant Sodium Dithionite and [FeFe] Hydrogenase: Influence on the Enzyme Mechanism
Source: J Am Chem Soc. 2021 Oct 20;143(43):18159–71. doi: 10.1021/jacs.1c07322 (PMC8569811; doi:10.1021/jacs.1c07322)
Supplement: Supplementary file 1 — ja1c07322_si_001.pdf [file ja1c07322_si_001.pdf]

Supporting Information

The non-physiological reductant sodium dithionite and [FeFe] hydrogenase: influence on the enzyme mechanism

Maria Alessandra Martini[1], Olaf Rüdiger[1], Nina Breuer[1], Birgit Nöring[1], Serena DeBeer[1], Patricia Rodríguez-Maciá[2], James A. Birrell[1*]*

[1]Department of Inorganic Spectroscopy, Max Planck Institute for Chemical Energy Conversion, Stiftstraße 34-36, 45470 Mülheim an der Ruhr, Germany

[2]Current address: Inorganic Chemistry Laboratory, Department of Chemistry, University of Oxford, South Parks Road, Oxford, OX1 3QR, UK

Corresponding Author

*james.birrell@cec.mpg.de
*patricia.rodriguezmacia@chem.ox.ac.uk

Contents

Experimental Section
Supplementary Discussion: Origins of $H_{ox}H$ and H_{red}'H
Table S1 | IR bands in [FeFe] hydrogenase states observed in this study
Table S2 | Comparison of IR bands in [FeFe] hydrogenase in the H_{ox} *vs* H_{ox}-DT_i and $H_{hyd\text{-}red}$ vs H_{hyd} states
Table S3 | Parameters used for pseudo-Voigt fitting
Figure S1 | Effect of oxNaDT on *Cr*HydA1^{ADT} at pH 7
Figure S2 | Effect of Na_2SO_4 and $Na_2S_2O_3$ on *Cr*HydA1^{ADT}
Figure S3 | Formation of the $H_{ox}H$ state in *Dd*HydAB and *Cp*HydA1 with Na_2SO_3 and low pH
Figure S4 | Full titration of *Cr*HydA1^{PDT} with Na_2SO_3 at pH 6 and pH 7 under N_2
Figure S5 | Titration of *Cr*HydA1^{PDT} with Na_2SO_3 at pH 4 and pH 5 under N_2
Figure S6 | Titration curves of *Cr*HydA1^{PDT}
Figure S7 | Speciation of SO_3^{2-} as a function of pH monitored by IR spectroscopy
Figure S8 | Full titration of *Cr*HydA1^{PDT} with Na_2SO_3 under 2% H_2 at pH 6
Figure S9 | Full titration of *Cr*HydA1^{PDT} with Na_2SO_3 under 2% H_2 at pH 7
Figure S10 | Full titration of *Cr*HydA1^{PDT} with Na_2SO_3 under 2% H_2 at pH 8 and pH 5
Figure S11 | Calculation of the redox potential of H_{ox}/H_{red} and $H_{ox}H/H_{red}$'H
Figure S12 | Formation of the $H_{ox}H$-CO state
Figure S13 | Cyclic voltammetry of *Dd*HydAB in the presence of Na_2SO_3
Figure S14 | Cyclic voltammetry of Na_2SO_3 at various pH values
Figure S15 | Cyclic voltammetry of *Dd*HydAB at various pH values

Figure S16 | Cyclic voltammetry of *Dd*HydAB at various pH values in the presence of Na_2SO_3
Figure S17 | Chronoamperometry experiments of *Dd*HydAB in the absence of Na_2SO_3
Supplementary Discussion: pH dependent redox potentials
Figure S18 | Model for pH dependent redox potential titrations of *Cr*HydA1^{PDT}

Experimental Section

Protein production

Sodium dithionite (NaDT) was excluded from all protein preparations to avoid contamination by its oxidation products. *Cr*HydA1, *Dd*HydAB and *Cp*HydA1 were recombinantly produced in *E. coli* as apo-proteins (lacking the $[2Fe]_H$ subcluster). Samples were then artificially maturated with the $[2Fe]_H$ precursor $(Et_4N)_2[Fe_2(ADT)(CO)_4(CN)_2]$ (using a ^{57}Fe-labeled version for NRVS experiments) or $(Et_4N)_2[Fe_2(PDT)(CO)_4(CN)_2]$, as previously described[1-3]. $(Et_4N)_2[Fe_2(ADT)(CO)_4(CN)_2]$ and $(Et_4N)_2[Fe_2(ADT)(CO)_4(CN)_2]$ were prepared as previously described[4, 5].

Sample preparation for FTIR Spectroscopy

Concentrated (2-5 mM) protein samples (in 25 mM Tris-HCl, 25 mM KCl, pH 8) were diluted (1 μL in 12 μL total volume) in 20 mM Na-acetate, 20 mM MES, 20 mM HEPES, 20 mM Tris, 20 mM glycine buffer (20 mM mixed buffer) adjusted to the desired pH with NaOH or HCl. Na_2SO_3, Na_2SO_4, $Na_2S_2O_3$ (all prepared in 20 mM mixed buffer) or oxidized NaDT (oxNaDT, prepared in water) were added at various concentrations (by substituting a fraction of the total 12 μL volume of the FTIR sample with a given volume of stock solution) as specified for each experiment. All solutions, except oxNaDT for some experiments where stated, were pH corrected before use. The solution of oxNaDT was prepared under aerobic conditions using fresh NaDT (1 M) and was left stirring at room temperature for 2 h under atmospheric oxygen to fully oxidize the dithionite anion. Before being added to the protein samples, the solution was degassed and moved into an anaerobic glovebox filled with N_2. To prepare *Cr*HydA1 in the H_{ox}-CO and H_{ox}H-CO states, samples (with or without Na_2SO_3) were flushed with 100% CO gas for 10 min before being loaded into the FTIR cell. For all Na_2SO_3 solutions, 1 M stock solutions were prepared in 20 mM mixed buffer, aerobically, and adjusted to the desired pH.

FTIR Spectroscopy

FTIR spectra were measured using a Bruker Vertex 80v FTIR spectrometer equipped with a mercury cadmium telluride detector cooled by liquid nitrogen. Spectra were recorded in the double-sided, forward-backward mode, with a resolution of 2 cm^{-1}, an aperture setting of 3 mm and scan velocity of 20 kHz. Spectra are the average of 100 scans. Samples (12 μL) were sealed between CaF_2 windows (20 mm x 4 mm, Korth Kristalle, Altenholz) separated by a 50 μm Teflon spacer coated with vacuum grease and closed in a homebuilt IR cell with rubber rings. FTIR data were processed using home-written routines in the MATLAB environment. For *Cr*HydA1^{PDT} prepared under 2% H_2, the region between 1955–1920 cm^{-1} (corresponding to the terminal CO on Fe_d) in each spectrum was fitted with pseudo-Voigt functions using QSoas 3.0 (ref [6]) to the identify the individual contributions from the H_{ox}, H_{red}, H_{ox}H and H_{red}'H states to the FTIR absorbance. Fit parameters (Table S3) were obtained for H_{ox} and H_{red} from spectra in the absence of Na_2SO_3. Parameters for H_{ox}H and H_{red}'H were obtained from the analysis of spectra at low pH and high Na_2SO_3 concentrations, where these were the dominant states, and by fixing the already determined parameters for H_{ox} and H_{red}. Fit parameters for the four states were then fixed in all subsequent fits (Table S3), allowing only the peak intensity to vary. Good agreement between simulated and experimental data was obtained for all spectra.

NRVS

NRVS spectra for $[2^{57}Fe]_H$-*Cr*HydA1 in 20 mM mixed buffer pH 6, with or without 1 M Na_2SO_3, were recorded at P01, PETRA-III, Hamburg.[7] The operation of the storage ring was a 40-bunch mode (bunch separation of 192 ns) with a beam energy of 6 GeV. The experimental setup established at the beamline including a two-step monochromatization (energy resolution of ∼ 1 meV) and detection by avalanche photo diodes was used.[7] Samples, in home-built copper samples holders, were positioned in the beam on the top of a closed cycle helium cryostat with a copper cold finger, using a home-built copper adapter. The sample was then covered with a mylar cylinder and a metal cover with a beryllium window. The temperature at the

base of the sample was maintained at 10 K and the Stokes/anti-Stokes imbalance derived real sample temperatures were 10–20 K. The NIS data were collected during several scans within the energy range of −20 to 100 meV with a 0.25 meV step size and a measuring time of 5 s per energy step. Data were collected as the raw nuclear inelastic scattering (NIS) counts. Calculation of partial density of vibrational states (PVDOS) was performed using routines written in Python with a binning of 0.5 meV.

Electrochemistry

*Dd*HydAB was covalently attached to a pyrolytic graphite electrode (0.031 cm^2, homemade using pyrolytic graphite from Momentive Materials) modified with 4-nitrobenzenediazonium salt as described previously.[8] Once modified, the electrode was moved inside a glovebox filled with N_2 and subjected to 10 cycles of voltammetry from +0.241 to −0.659 V to remove oxygen adsorbed on the electrode surface. *Dd*HydAB was immobilized by covering the electrode with 6 µL of protein solution (10 µM in 10 mM MES pH 5.8). The protein was left to adsorb for 20 min. Subsequently, N-hydroxysuccinimide (NHS, 4.5 µL of 128 mM solution) and N-(3-dimethylaminopropyl)-N'-ethylcarbodiimide (EDC, 5.5 µL of 210 mM solution), both dissolved in 10 mM MES pH 5.8, were added to the electrode and left to react for 90 min. The electrode was rinsed with water and then in the appropriate experimental buffer by rotating the electrode inside the buffer solution for 1 min. Electrochemical measurements were performed in a standard three-electrode electrochemical cell with a platinum wire as counter electrode and a saturated calomel reference electrode, separated from the main compartment in a sidearm containing 0.1 M KCl, connected to the main cell compartment by a Luggin capillary. The reference electrode potential was calibrated using (hydroxymethyl)ferrocene (+436 mV vs SHE) as a reference.[9] The potential was controlled by a VersaSTAT 4–400 potentiostat. A set of mass flow-controllers was used to control the composition of the gas flushed through the electrochemical cell. A cell temperature of 25 °C was maintained by a waterjacket system and a water circulator. All the experiments were performed in 20 mM mixed buffer at the desired pH with addition of 0.1 M NaCl. All potentials are quoted versus the standard hydrogen electrode (SHE).

Supplementary Discussion: Origins of $H_{ox}H$ and H_{red}'H

As mentioned in the main text, the $H_{ox}H$ and H_{red}'H states in Model 2 have been reported to accumulate at low pH in the presence of sodium dithionite (NaDT) and were proposed to be protonated versions of H_{ox} and H_{red}', respectively, based on density functional theory (DFT) calculations.[10, 11] $H_{ox}H$, originally identified during studies of the H_{hyd} state, exhibits an infrared (IR) spectrum similar to that of H_{ox}, but with vibrational frequencies shifted higher in energy by 4-6 cm^{-1} (Table S1).[12] Stripp and coworkers suggested that the $H_{ox}H$ state is protonated on the sulfur of one of the cysteine residues ligating $[4Fe\text{-}4S]_H$,[11] the same cysteine proposed to be protonated in H_{red}',[10] which in Model 2 forms from H_{ox} via PCET at $[4Fe\text{-}4S]_H$. This protonation is thought to have a regulatory effect on catalysis, preventing the formation of the low-activity reduced states containing a μH^{-}.[13-15] Recently, the presence of a μH^{-} in the $H_{red}H^{+}$ and $H_{sred}H^{+}$ states has been disputed,[16-18] and the redox potential of $[4Fe\text{-}4S]_H$ has been shown to be pH independent,[19] challenging the proposed PCET at $[4Fe\text{-}4S]_H$ in the formation of H_{red}'.

As the reductant NaDT is needed for the formation of $H_{ox}H$, this state was proposed to lie at the end of the catalytic cycle: after H_{red}' and H_{hyd}, where the proton on $[4Fe\text{-}4S]_H$ is retained. Deprotonation of $H_{ox}H$, which would reform H_{ox}, is hindered at low pH, potentially explaining why this state accumulates under acidic conditions. However, it is unclear why the H_{ox} state does not simply convert to $H_{ox}H$ at low pH. The H_{red}'H state was reported to form upon reduction and protonation of $H_{ox}H$, under H_2 or via electrochemical reduction, but again only in the presence of NaDT at low pH.[10, 11] Similar to H_{ox} *vs* $H_{ox}H$, the infrared spectrum of H_{red}'H resembles the one of H_{red}', but with bands shifted to slightly higher energies (Table S1). DFT calculations suggested that, in H_{red}'H, a second proton, in addition to the one proposed to be already bound in $H_{ox}H$ and H_{red}', binds to another cysteine residue ligating the $[4Fe\text{-}4S]_H$ subcluster. Whether H_{red}'H is an intermediate of the catalytic cycle was not clarified. Importantly, both $H_{ox}H$ and H_{red}'H have not been reported to form with chemical reducing agents other than NaDT, including H_2, or via electrochemical reduction in the absence of NaDT. A satisfactory explanation for this behavior is still lacking.

Supplementary Tables

Table S1 – IR bands in [FeFe] hydrogenase states observed in this and previous studies

Enzyme	State[#]	CN_p	CN_d	CO_p	CO_d	CO_b	Ref.
*Cr*HydA1^{ADT}	H_{ox}	2089<br>2088	2072<br>2072	1964<br>1964	1940<br>1940	1804<br>1802	Here<br>Ref [11]
	$H_{ox}H$	2092<br>2092	2076<br>2076	1971<br>1971	1947<br>1946	1813<br>1812	Here<br>Ref [11]
	H_{red}<br>H_{red}’	2084<br>2084	2067<br>2066	1962<br>1962	1933<br>1933	1793<br>1792	Here<br>Ref [11]
	H_{red}’H	2086	2068	1966	1938	1800	Ref [11]
	H_{ox}-CO	2092<br>2091	2082<br>2081	1964<br>1962	2013/1969*<br>2012/1968*	1810<br>1808	Here<br>Ref [11]
	H_{ox}H-CO	2094<br>2094	2086<br>2086	1966<br>1966	2006/1972*<br>2006/1972*	1816<br>1816	Here<br>Ref [11]
*Cr*HydA1^{PDT}	H_{ox}	2090<br>2090	2073<br>2073	1966<br>1965	1942<br>1941	1811<br>1810	Here<br>Ref [11]
	$H_{ox}H$	2091<br>2090	2075<br>2075	1970<br>1969	1946<br>1945	1813<br>1814	Here<br>Ref [11]
	H_{red}<br>H_{red}’	2085<br>2084	2066<br>2066	1964<br>1963	1935<br>1934	1799<br>1798	Here<br>Ref [11]
	H_{red}’H	2084<br>2084	2068<br>2068	1966<br>1966	1939<br>1938	1802<br>1802	Here<br>Ref [11]
*Dd*HydAB^{ADT}	H_{ox}	2087<br>2090	2078<br>2078	1965<br>1965	1940<br>1940	1802<br>1802	Here<br>Ref [11]
	$H_{ox}H$	2096<br>2098	2082<br>2083	1972<br>1972	1947<br>1947	1811<br>1812	Here<br>Ref [11]
*Cp*HydA1^{ADT}	H_{ox}	2082<br>2081	2070<br>2069	1970<br>1970	1947<br>1947	1801<br>1801	Here<br>Ref [11]
	$H_{ox}H$	2084<br>2085	2073<br>2074	1975<br>1975	1953<br>1953	1810<br>1808	Here<br>Ref [11]

when two nomenclatures are given, the one underneath is from Stripp and Haumann[20]

*In the H_{ox}-CO and H_{ox}H-CO states the two CO ligands bound to Fe_d are strongly vibrationally coupled giving rise to a higher energy symmetric stretching band and a lower energy antisymmetric stretching band.

Table S2 – Comparison of IR bands in [FeFe] hydrogenase in the H_{ox} *vs* H_{ox}-DT_i and $H_{hyd:red}$ vs H_{hyd} states

Enzyme	State	CN_p	CN_d	CO_p	CO_d	CO_b	Ref.
*Cr*HydA1^{ADT}	H_{ox}	2089<br>2088	2072<br>2072	1964<br>1964	1940<br>1940	1804(2)<br>1802	Here<br>Ref [11]
	H_{ox}-DT_i	2092<br>2092	2076<br>2074	1971<br>1970	1947<br>1946	1813<br>1812	Here<br>Ref [11]
	$H_{hyd:ox}$	2092	2086	1983	1954	1865	Ref [18]
	$H_{hyd:red}$	2087	2078	1972	1954	1851	Ref [18]
	H_{hyd}	2088	2076	1980	1960	1860	Ref [12]

Table S3 – Parameters used for Pseudo-Voigt fitting

	Pseudo-Voigt fitting parameters		
**State**	$\mathbf{x_i}$	$\mathbf{w_i}$	$\boldsymbol{\mu_i}$
H_{ox}	1942.08	1.79	0.426
H_{red}	1935.23	2.36	0.535
$H_{ox}H$	1946.15	1.83	0.546
H_{red}'H	1938.67	2.76	0.705

Where x_i, w_i and μ_i are parameters for each of the four pseudo-Voigt peaks fit to the following formula, while A_i was allowed to vary:

$$f(x) = Y_0 + \sum_i A_i \left[\frac{1-\mu_i}{\sqrt{2\pi w_i^2}} \exp(-(x-x_i)^2/2w_i{}^2) + \frac{\mu_i}{w_i \pi (1 + ((x-x_i)/w_i)^2)} \right]$$

x_i = peak center, w_i = broadening factor, μ_i = profile shape factor

Supplementary Figures

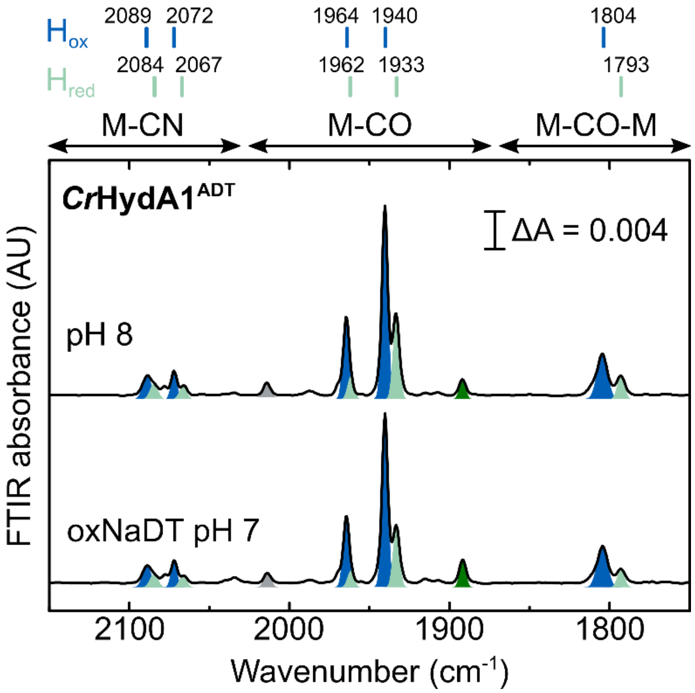

Figure S1 – Effect of oxNaDT on *Cr*HydA1^{ADT} at pH 7. IR spectra of *Cr*HydA1 (≈730 µM) diluted in 25 mM Tris-HCl, 25 mM NaCl, pH 8 (top) and in a solution of oxidized NaDT (0.83 M final concentration) corrected to pH 7 (bottom). At neutral pH the H_{ox}H state does not form in the presence of the oxidation products of NaDT.

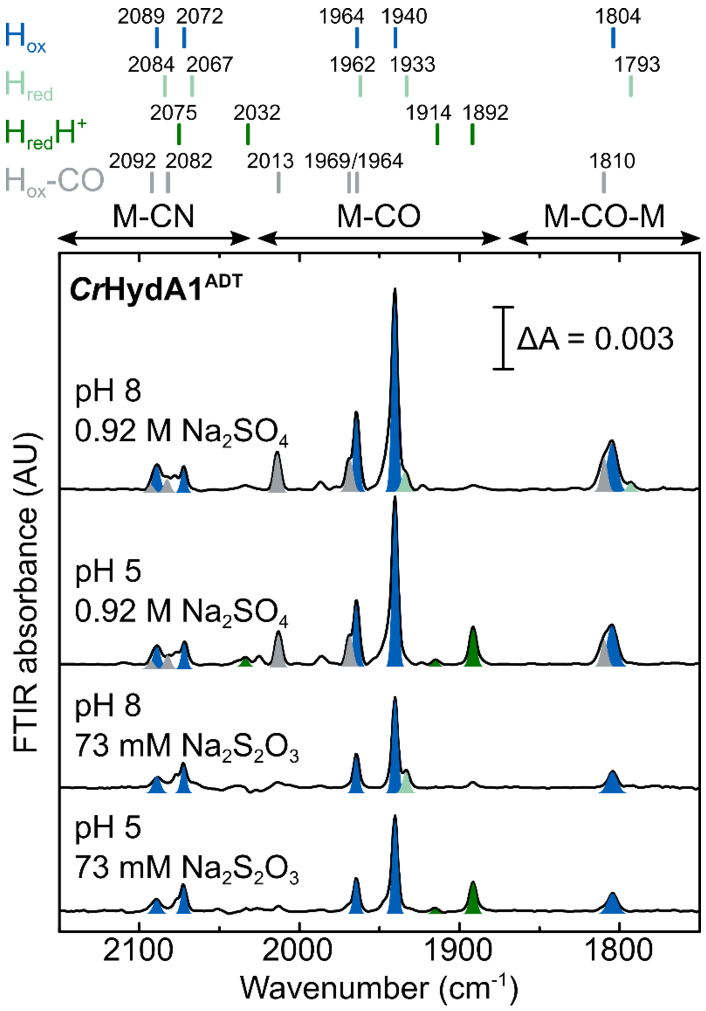

Figure S2 – Effect of Na_2SO_4 and $Na_2S_2O_3$ on *Cr*HydA1^{ADT}. IR spectra of *Cr*HydA1 (0.2 mM) diluted in 20 mM mixed buffer (see experimental section) at pH 8 and pH 5 in the presence of 0.92 M Na_2SO_4 or 73 mM $Na_2S_2O_3$.

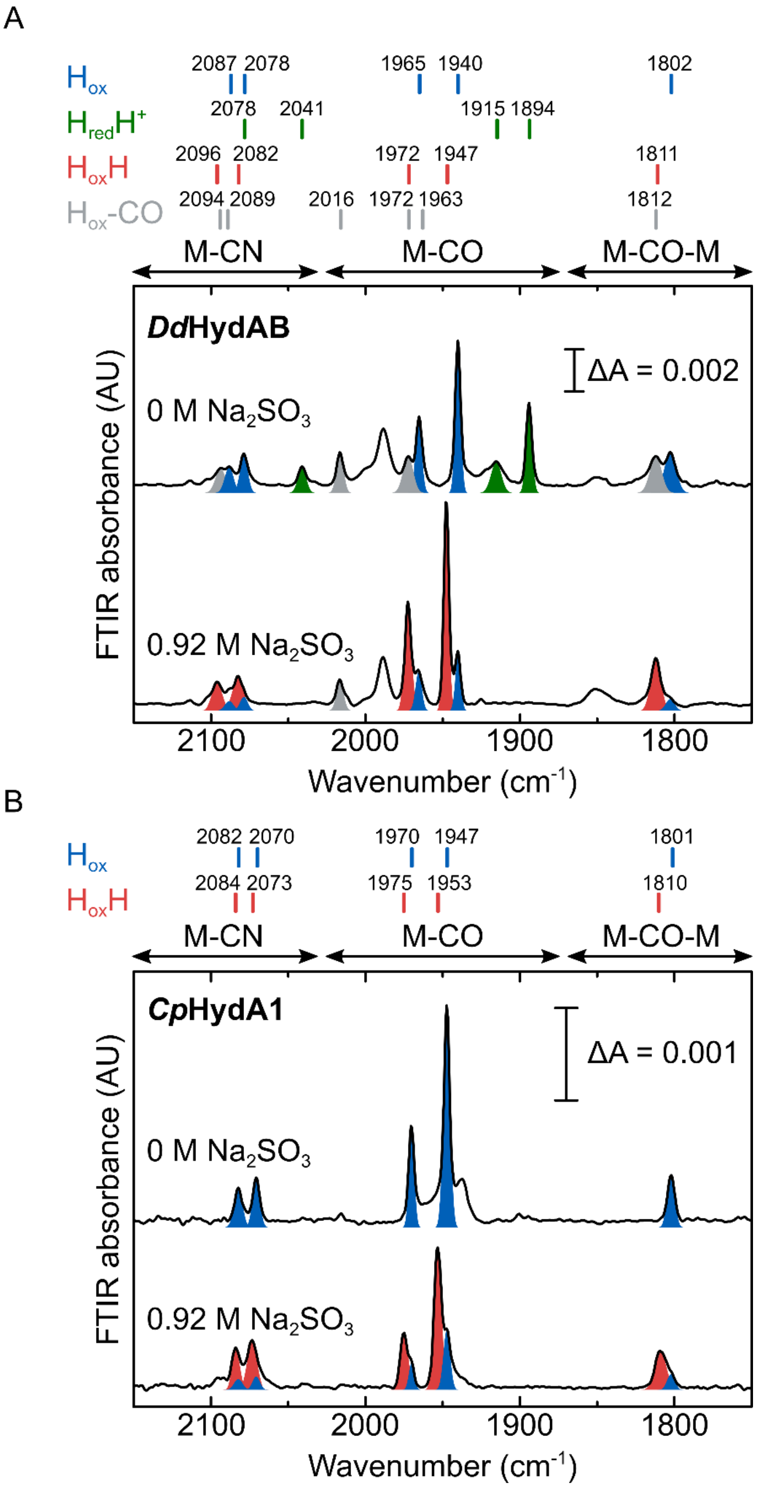

Figure S3 – Formation of the $H_{ox}H$ state in *Dd*HydAB and *Cp*HydA1 with Na_2SO_3 and low pH. A) IR spectra of *Dd*HydAB (≈250 µM) in 20 mM mixed buffer (see experimental section), pH 6 (top) and in 0.92 M Na_2SO_3 pH 6 (bottom). **B**) Same as **A** but with *Cp*HydA1 (≈60 µM). The unassigned peaks at 1988 cm^{-1} and 1950 cm^{-1} in the *Dd*HydAB spectra are often observed for this enzyme and appear to be related to the longer artificial maturation process compared with *Cp*HydA1 and *Cr*HydA1. Further investigation of the origin of these peaks will be the subject of ongoing investigation.

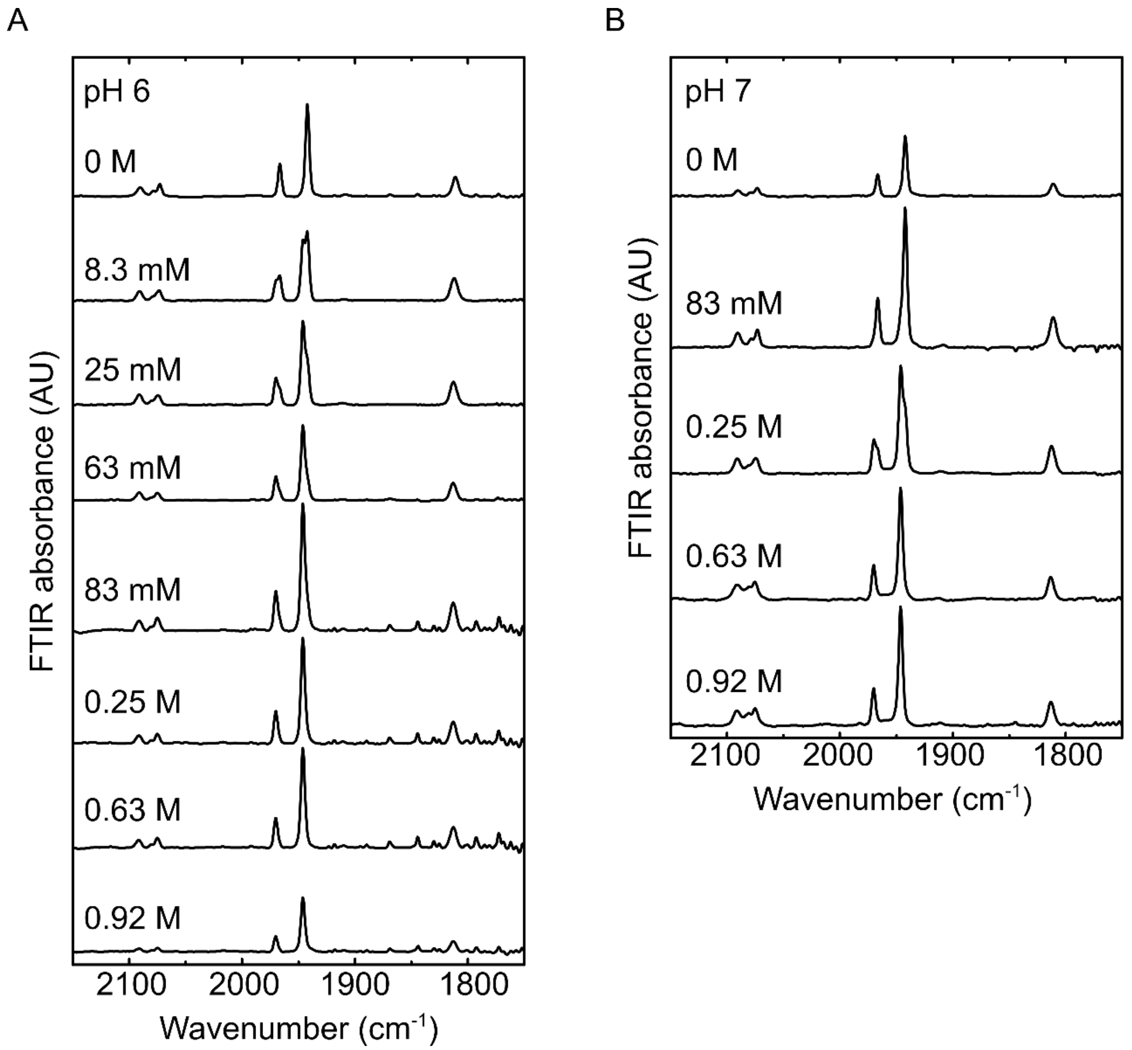

Figure S4 – Full titration of *Cr*HydA1^{PDT} with Na_2SO_3 at pH 6 and pH 7 under N_2. IR spectra of *Cr*HydA1^{PDT} (≈420 µM) diluted in 20 mM mixed buffer (see experimental section) at pH 6 (**A**) and pH 7 (**B**) with various concentrations of Na_2SO_3. Selected spectra are shown in **Figure 3** (main text).

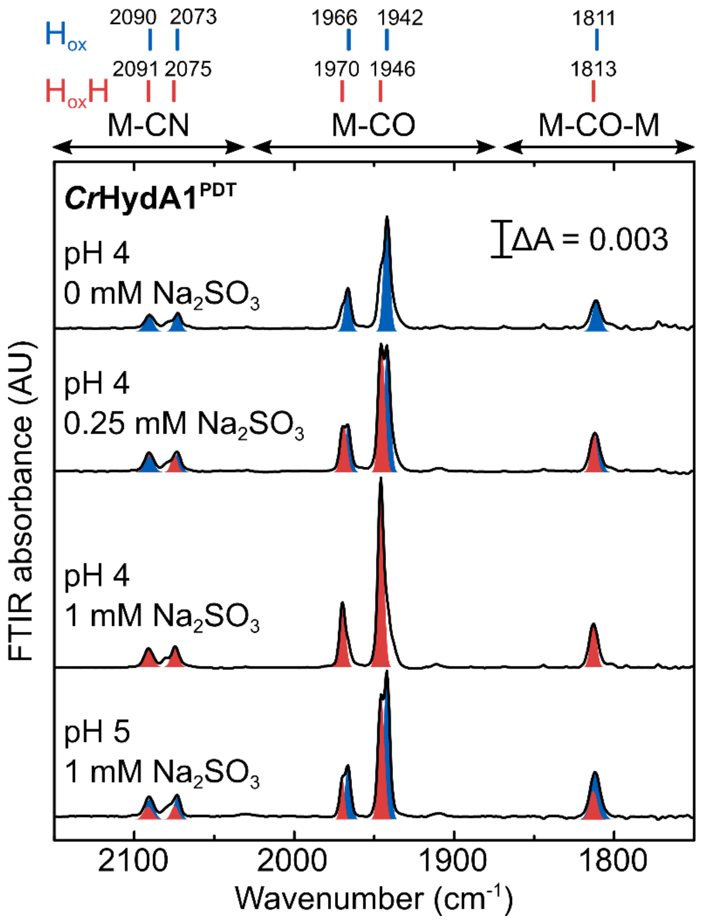

Figure S5 – Titration of *Cr*HydA1^{PDT} with Na_2SO_3 at pH 4 and pH 5 under N_2. IR spectra of *Cr*HydA1^{PDT} (≈420 µM) diluted in 20 mM mixed buffer (see experimental section) at pH 4 and pH 5 with various concentrations of Na_2SO_3.

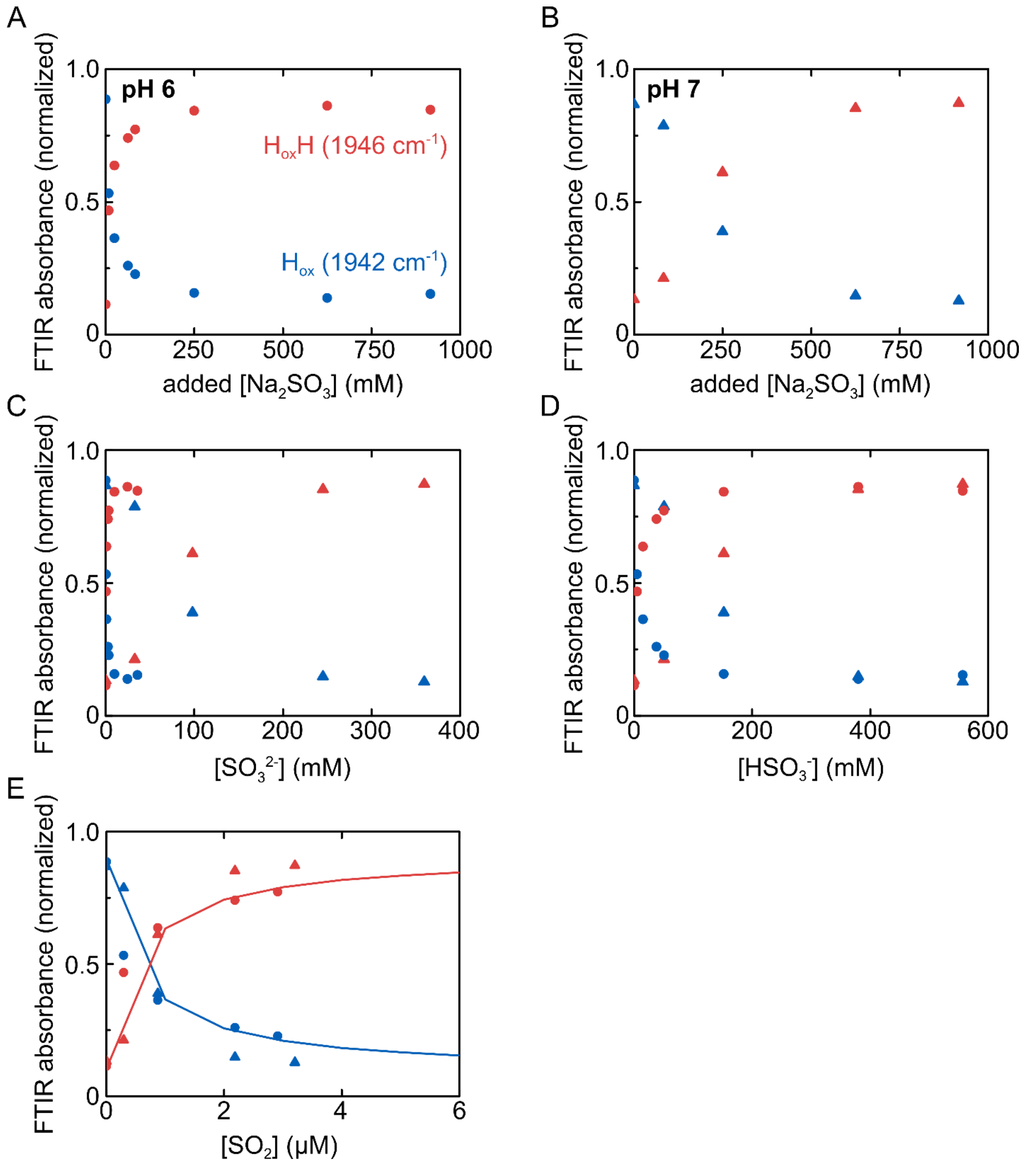

Figure S6 – Titration curves of *Cr*HydA1^{PDT}. A, **B**) Variation in the intensity of the 1942 cm^{-1} (H_{ox}) and 1946 cm^{-1} ($H_{ox}H$) peaks with the concentration of added Na_2SO_3 at pH 6 (**A**) and pH 7 (**B**). **C**, **D**) as in **A** and **B**, but the data (triangles for pH 7, circles for pH 6) are plotted as a function of the estimated concentration of SO_3^{2-} (**C**) and HSO_3^- (**D**). **E**) Expanded version of the region from 0 to 6 μM SO_2 in **Figure 3C** (main text).

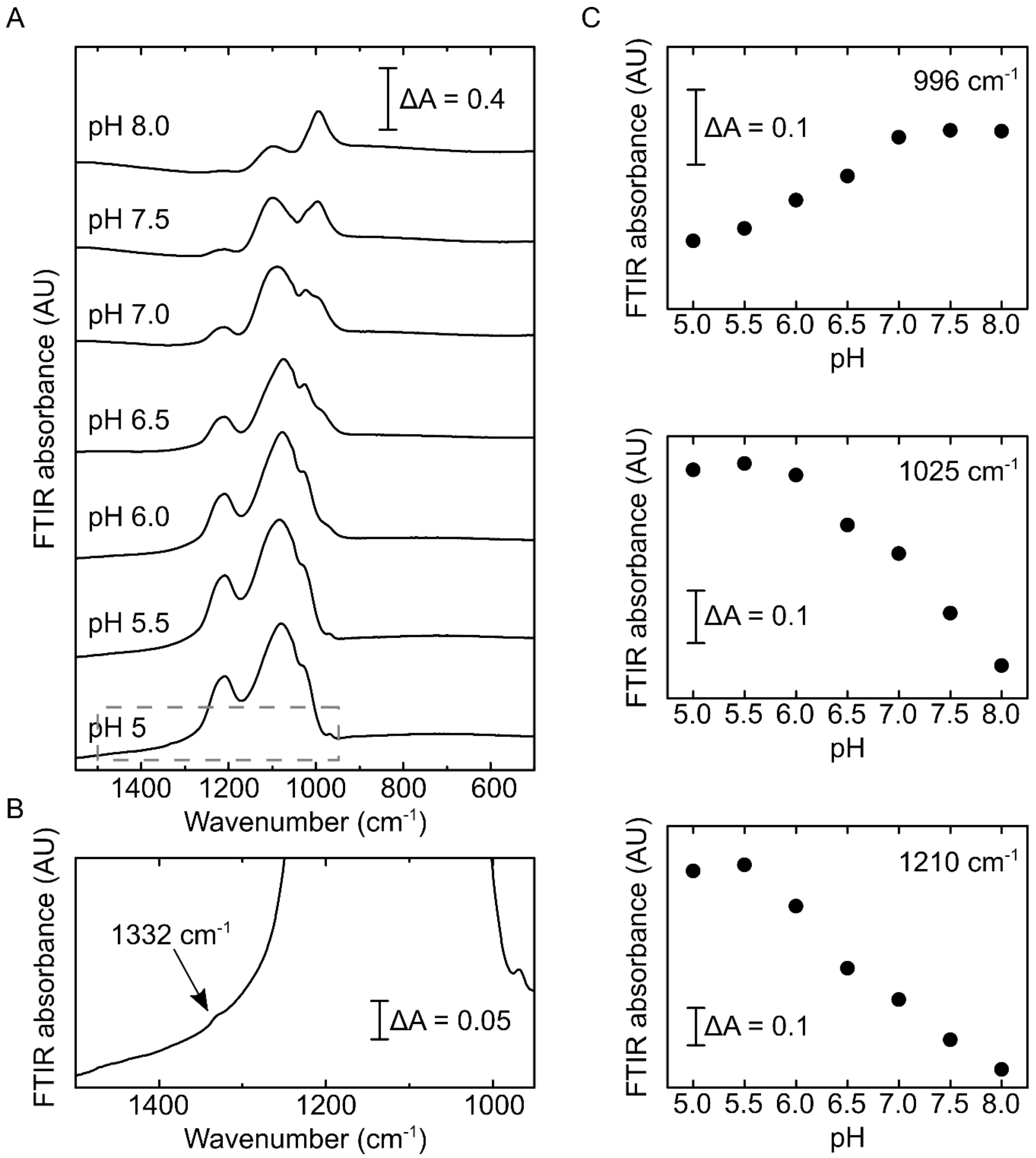

Figure S7 – Speciation of SO_3^{2-} as a function of pH monitored by IR spectroscopy. A) Spectra of Na_2SO_3 (1 M) at different pH values. **B**) Enlargement of the pH 5 spectrum (dashed rectangle in **A**), indicating the presence of dissolved SO_2 (ν_3 band at 1332 cm^{-1}, ref[21]). **C**) Titration of the features at 996 cm^{-1}, 1025 cm^{-1}, 1210 cm^{-1} with pH. The feature at 996 cm^{-1} was assigned to SO_3^{2-}, while the ones at 1025 cm^{-1}, 1210 cm^{-1} to HSO_3^- or one of its dimers as reported in ref [21].

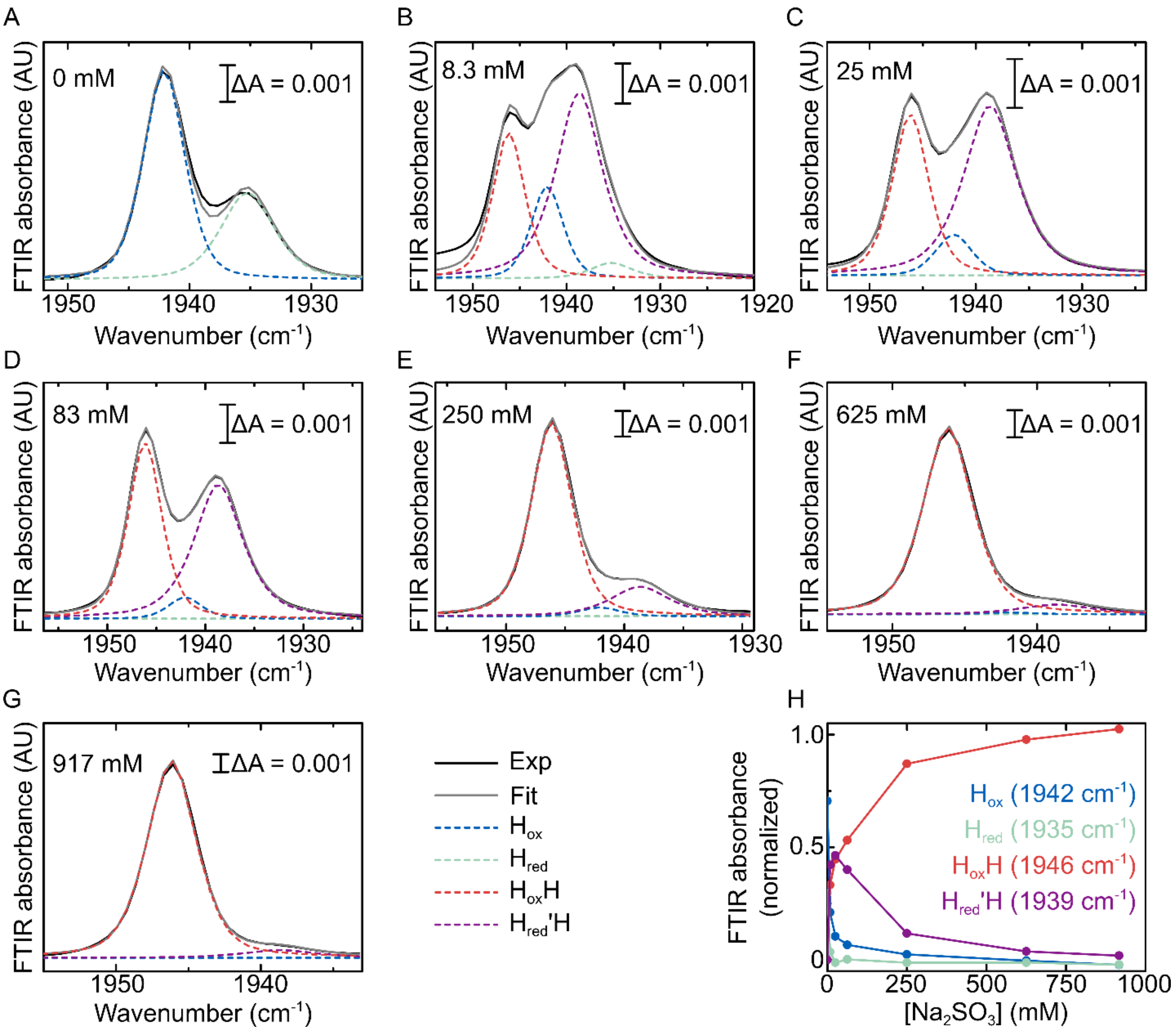

Figure S8 – Full titration of *Cr*HydA1^{PDT} with Na_2SO_3 under 2% H_2 at pH 6. A-G) Pseudo-Voigt peak-fitting analysis of the 1955-1920 cm^{-1} in IR spectra of *Cr*HydA1^{PDT} measured under 2% H_2 at pH 6. **H**) Variation in the intensity of the 1942 cm^{-1} (H_{ox}), 1935 cm^{-1} (H_{red}), 1946 cm^{-1} ($H_{ox}H$) and 1939 cm^{-1} (H_{red}'H) peaks from **A-G** with the Na_2SO_3 concentration at pH 6. The lines connecting the points in **H** are for visual purposes only.

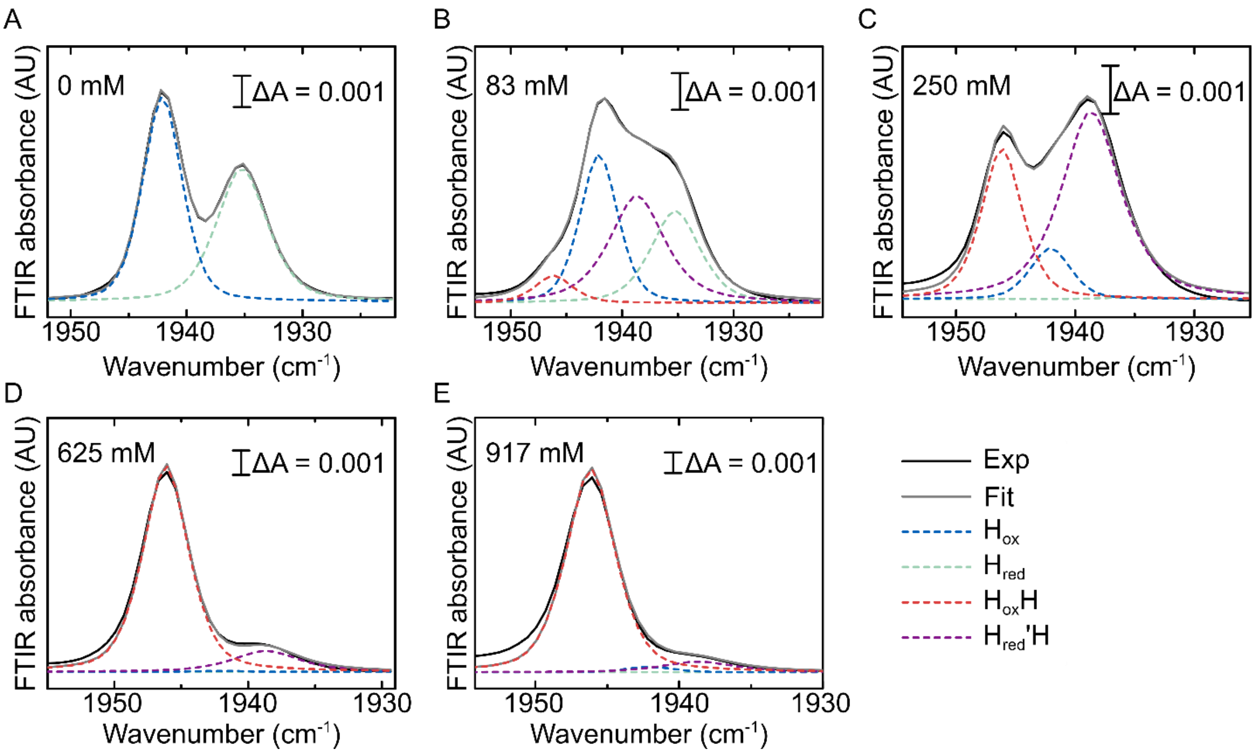

Figure S9 – Full titration of *Cr*HydA1^{PDT} with Na_2SO_3 under 2% H_2 at pH 7. A-E) Pseudo-Voigt peak-fitting analysis of the 1955-1920 cm^{-1} in IR spectra of *Cr*HydA1^{PDT} measured under 2% H_2 at pH 7. Data in panel **B** is also shown in **Figure 4A** (main text).

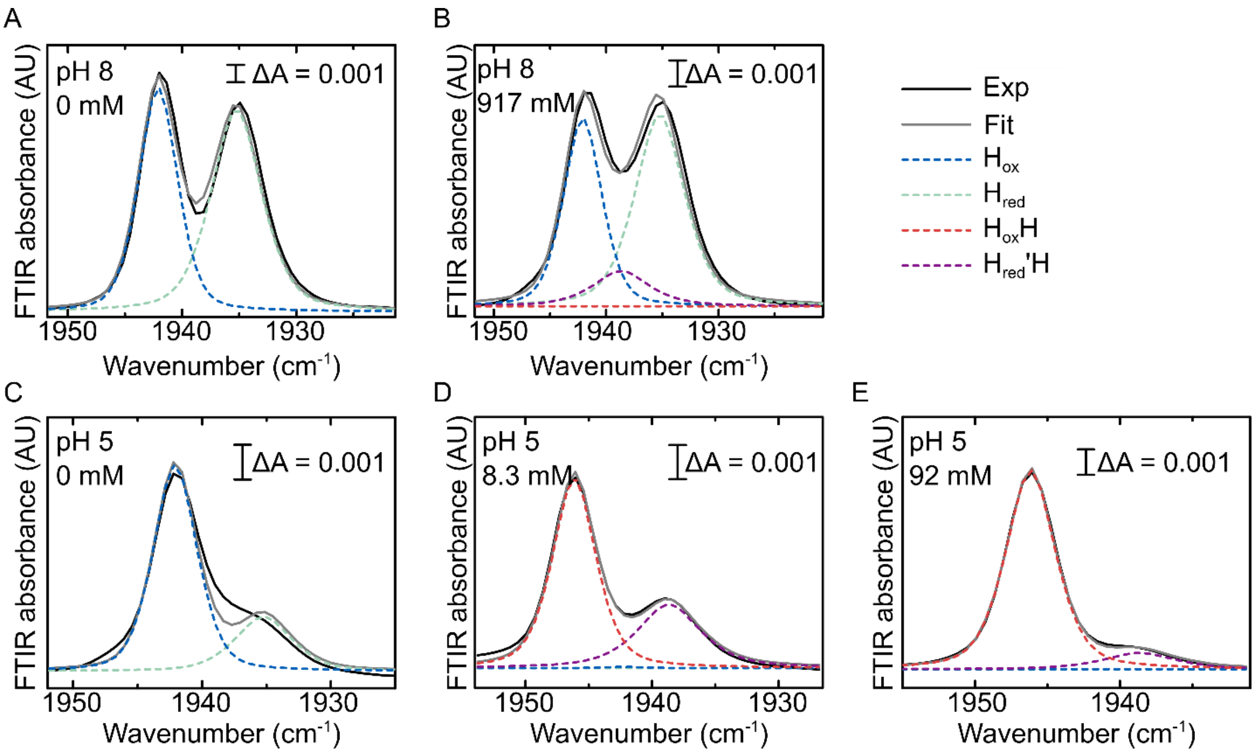

Figure S10 – Full titration of *Cr*HydA1^{PDT} with Na_2SO_3 under 2% H_2 at pH 8 and pH 5. Pseudo-Voigt peak-fitting analysis of the 1955-1920 cm^{-1} in IR spectra of *Cr*HydA1^{PDT} measured under 2% H_2 at pH 8 (**A**, **B**) and 5 (**C**, **D**, **E**).

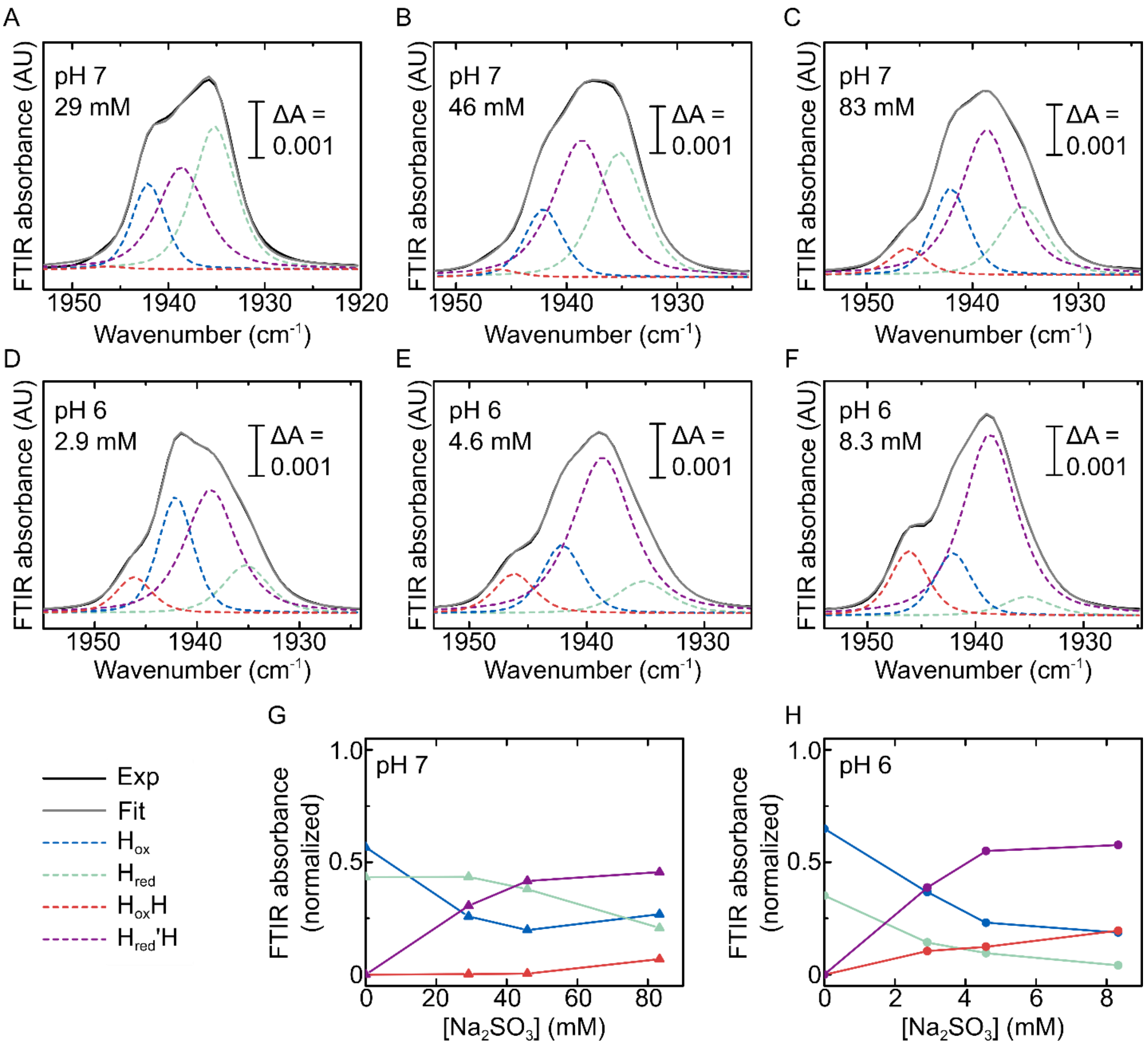

Figure S11 – Calculation of the redox potential of H_{ox}/H_{red} and $H_{ox}H/H_{red}$'H. Pseudo-Voigt peak-fitting analysis of the 1955-1920 cm^{-1} of additional IR spectra of *Cr*HydA1^{PDT} measured under 2% H_2 at pH 7 (**A-C**) and 6 (**D-F**) at low concentrations of sulfite. **G**, **H**) Variation in the intensity of the 1942 cm^{-1} (H_{ox}, blue), 1935 cm^{-1} (H_{red}, cyan), 1946 cm^{-1} (H_{ox}H, red) and 1939 cm^{-1} (H_{red}'H, purple) peaks with the Na_2SO_3 concentration at pH 7 (**G**, data from **A-C**) and at pH 6 (**H**, data from **D-F**). The lines connecting the points in **G**, **F** are for visual purposes only. At low Na_2SO_3 concentrations, all four state (H_{ox}, H_{red}, H_{ox}H and H_{red}'H) are present at the same time in most cases. At higher concentrations, oxidation of H_2 by the added Na_2SO_3 becomes more prominent, potentially causing an overestimation of the redox potentials. The redox potentials can be calculated for each condition from the H_{ox}/H_{red} and $H_{ox}H/H_{red}$'H ratios using the Nernst equation and adjusting the $2H^+/H_2$ redox potential accoring to the pH (7 or 6) – for more details see ref[19]. Data in **A** and **B** were not used to determine the $H_{ox}H/H_{red}$'H redox potential, as the H_{ox}H state is not present (see also **G**). Data from **Figure S8B** and **Figure S9B** were included in the calculation of both H_{ox}/H_{red} and $H_{ox}H/H_{red}$'H redox potentials. The redox potentials were calculated to be E_m (H_{ox}/H_{red}) = –349 (± 17) mV and E_m ($H_{ox}H/H_{red}$'H) = –293 (± 26) mV (mean ± standard deviation, based on the low Na_2SO_3 datapoints at pH 6 and 7).

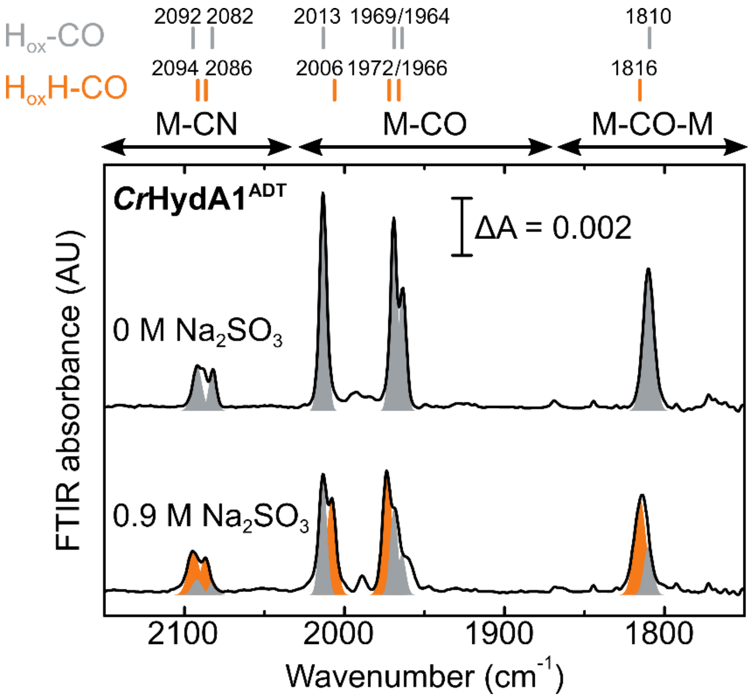

Figure S12 – Formation of the H_{ox}H-CO state. IR spectra of *Cr*HydA1[ADT] (≈360 µM) diluted in 20 mM mixed buffer at pH 5 (top) or in 0.92 M Na_2SO_3 at pH 5 (bottom) and flushed with 100% CO gas for 10 min before measuring the spectra.

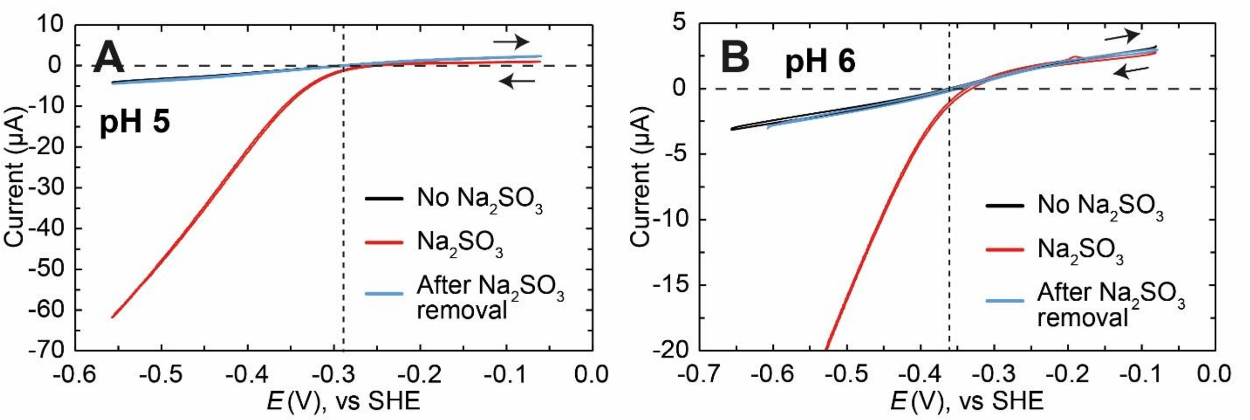

Figure S13 – Cyclic voltammetry of *Dd*HydAB in the presence of Na_2SO_3. Enlarged versions of the CVs reported in **Figure 6A** (**A**, pH 5) and **Figure 6B** (**B**, pH 6) in the main text. The black arrows indicate the scan direction of the CV. The dashed horizontal line shows the zero current position and the dashed vertical line shows the equilibrium $2H^+/H_2$ potential at each pH value.

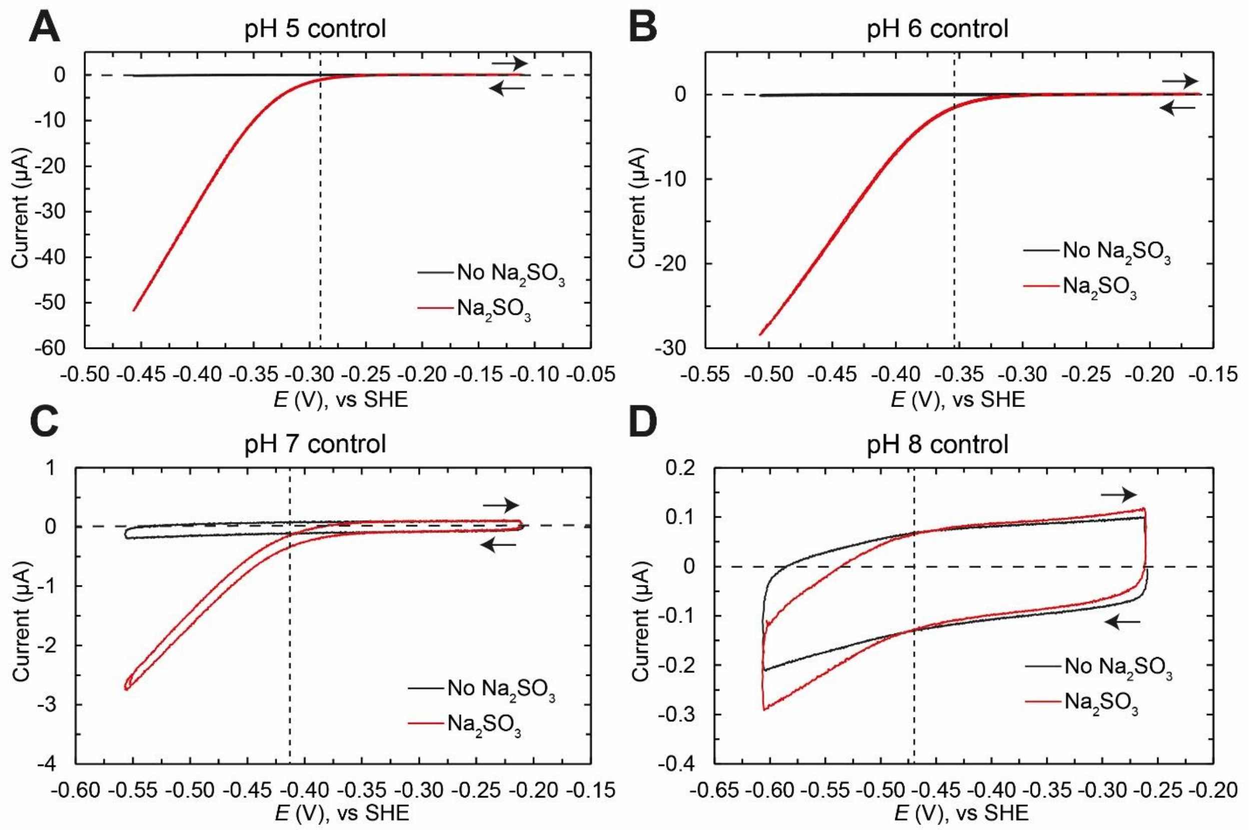

Figure S14 – Cyclic voltammetry of Na_2SO_3 at various pH values. A blank PGE electrode was used for cyclic voltammetry in 20 mM mixed buffer containing 100 mM NaCl at pH 5 (**A**), 6, (**B**), 7 (**C**) and 8 (**D**) in the absence (black curve) and presence (red curve) of 40 mM Na_2SO_3. Cyclic voltammetry was measured at 25 ^oC with 100% H_2 flow and 2000 rpm rotation rate. The black arrows indicate the scan direction of the CV. The dashed horizontal line shows the zero current position and the dashed vertical line shows the equilibrium $2H^+/H_2$ potential at each pH value.

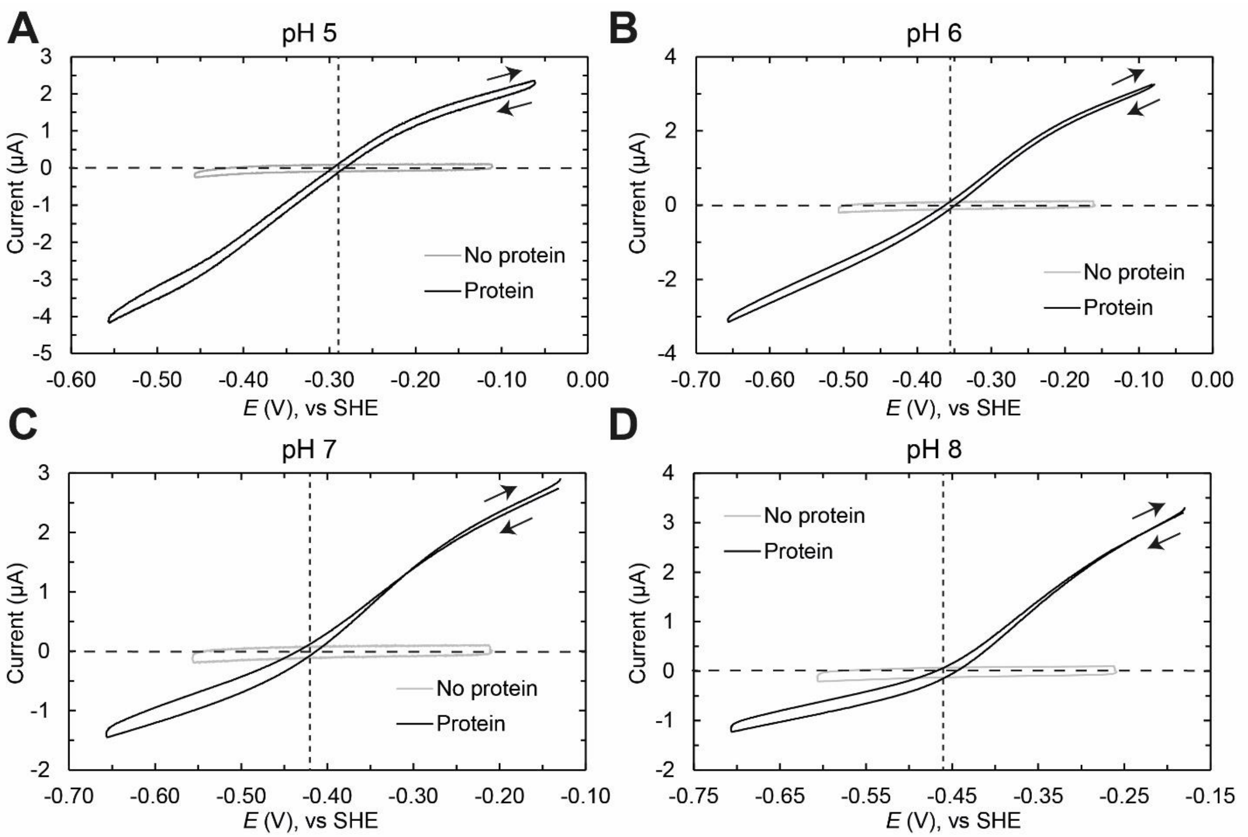

Figure S15 – Cyclic voltammetry of *Dd*HydAB at various pH values. A blank (gray curves) PGE electrode and a *Dd*HydAB modified PGE electrode (black curves) were used for cyclic voltammetry in 20 mM mixed buffer containing 100 mM KCl at pH 5 (**A**), 6, (**B**), 7 (**C**) and 8 (**D**). Cyclic voltammetry was measured at 25 ºC with 100% H_2 flow and 2000 rpm rotation rate. The black arrows indicate the scan direction of the CV. The dashed horizontal line shows the zero current position and the dashed vertical line shows the equilibrium $2H^+/H_2$ potential at each pH value.

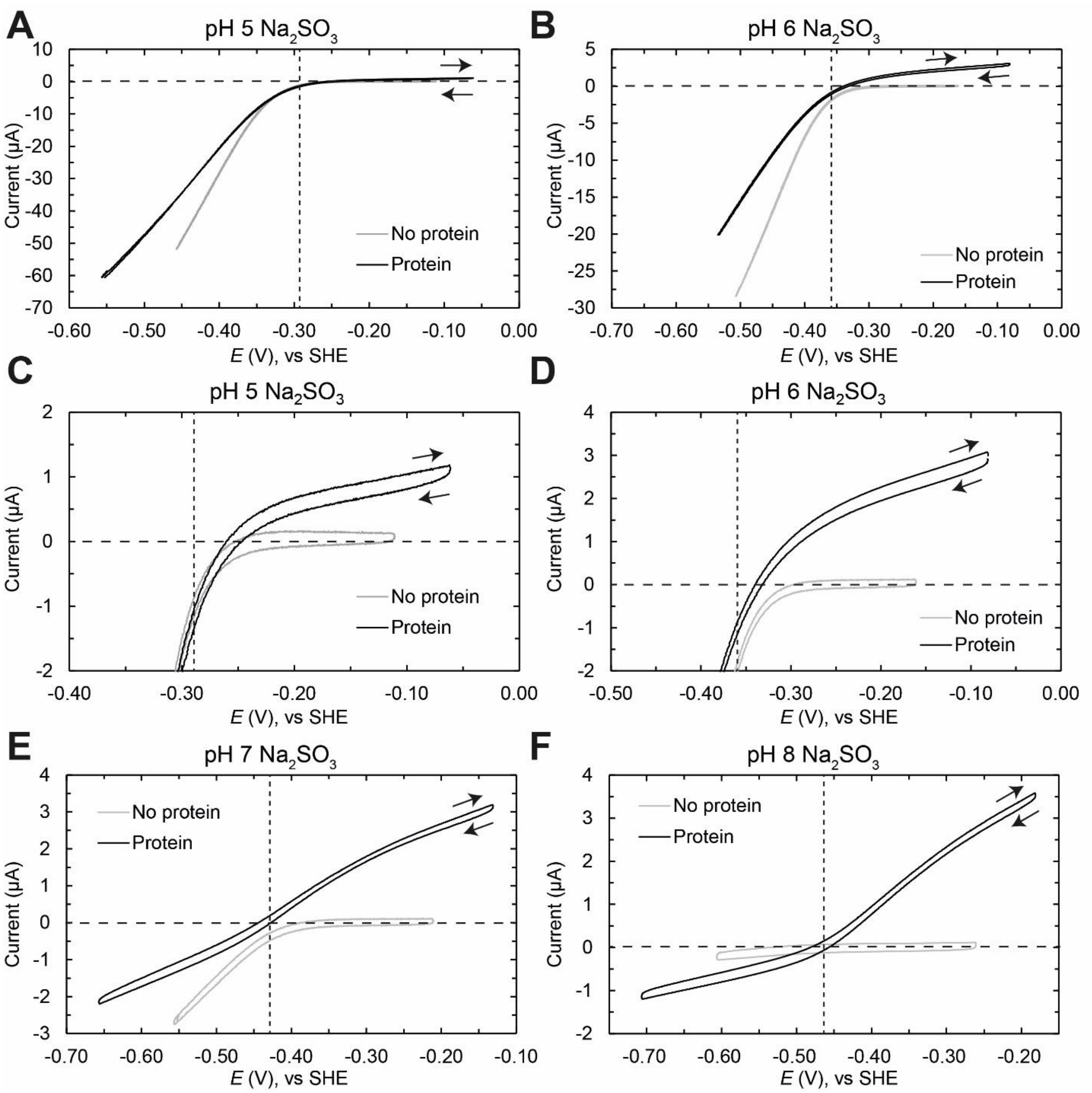

Figure S16 – Cyclic voltammetry of *Dd*HydAB at various pH values in the presence of Na_2SO_3. A blank (gray curves) PGE electrode and a *Dd*HydAB modified PGE electrode (black curves) were used for cyclic voltammetry in 20 mM mixed buffer containing 100 mM KCl and 40 mM Na_2SO_3 at pH 5 (**A**), 6, (**B**), 7 (**E**) and 8 (**F**). Enlarged versions of the H_2 oxidation region of **A** and **B** are shown in **C** and **D**, respectively. Cyclic voltammetry was measured at 25 °C with 100% H_2 flow and 2000 rpm rotation rate. The black arrows indicate the scan direction of the CV. The dashed horizontal line shows the zero current position and the dashed vertical line shows the equilibrium $2H^+/H_2$ potential at each pH value.

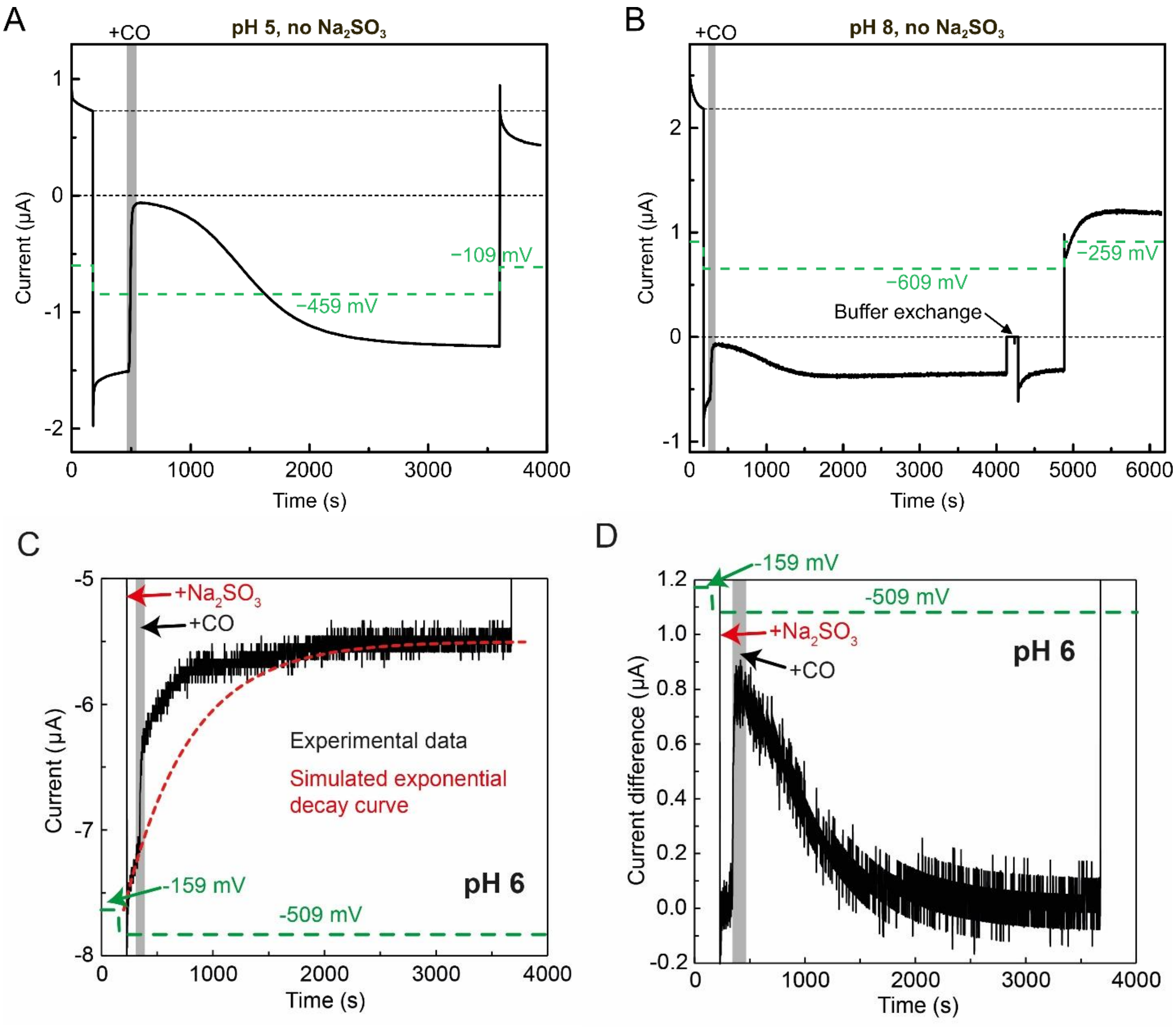

Figure S17 – Chronoamperometry experiments of *Dd*HydAB in the absence of Na_2SO_3. A *Dd*HydAB modified PGE electrode was used for chronoamperometry experiments in 20 mM mixed buffer containing 100 mM NaCl at pH 5 (**A**) and 8 (**B**) under 1 bar 90 % H_2 in N_2 (1000 ml/min), at 25 °C and with 2000 rpm rotation. The potential was varied as indicated by the green profile, where all potentials are reported *vs* SHE. At pH 5 (**A**), the potential was set initially to -109 mV, then to -459 mV and finally to -109 mV. At pH 8 (**B**) it was set initially to -259 mV, then to -609 mV and finally to -259 mV. Addition of 10% CO to the gas mixture (replacing 10% N_2) is indicated by the shaded area. In **B** after 4000 s the buffer was exchanged to fresh buffer. (**C**) Expanded region of Figure 7B (pH 6 with Na_2SO_3) from the main text with a simulated exponential decay curve (red dashed line) overlaying the experimental data. (**D**) The current difference between the experimental data and the simulated exponential decay curve from **C** is plotted showing a positive increase in the current after addition of CO, followed by a decay of the difference back to 0. This behavior is analogous to that observed for the experiments in the absence of Na_2SO_3 (**A** and **B**).

Supplementary Discussion: pH dependent redox potentials

As mentioned in the main text, Senger *et al.* observed a pH dependent redox potential for both the H_{ox}/H_{red}' and $H_{ox}H/H_{red}$'H (their nomenclature) transitions in *Cr*HydA1^{PDT}, as well as a pH dependent formation of $H_{ox}H$ from H_{ox}.[10] They attributed this to protonation of the $[4Fe\text{-}4S]_H$ subcluster of the H-cluster at two distinct sites – one site was responsible for the pH dependence of the H_{ox}/H_{red} and $H_{ox}/H_{ox}H$ transitions, and the other site was responsible for the $H_{ox}H/H_{red}$'H and H_{red}/H_{red}'H transitions. However, as we mentioned in a previous report,[19] simply having a proton-dependent step linking H_{ox} and $H_{ox}H$ as well as H_{red}' and H_{red}'H gives rise to apparent pH dependencies for all of the above-mentioned transitions. As such, two protonation sites are not required. We also demonstrated that, in the absence of sodium dithionite, neither $H_{ox}H$ nor H_{red}'H are observed.[19] This behavior is not in agreement with a simple protonation of the [4Fe-4S] cluster.

In the current work, we have shown that the formation of $H_{ox}H$ and H_{red}'H are strictly dependent on Na_2SO_3, an oxidation product of sodium dithionite. Moreover, these two states only form with Na_2SO_3 at low pH implicating SO_2 as the species that interacts with the H-cluster to form $H_{ox}H$ and H_{red}'H. Accordingly, we have renamed these states as H_{ox}-DT_i and H_{red}-DT_i (for dithionite inhibited). With this knowledge at hand we can try and explain the redox titrations published by Senger *et al.* In Figure S18, theoretical redox potential titrations of *Cr*HydA1^{PDT} can be observed for pH 7.5 (Figure S18A), pH 6.5 (Figure S18B) and pH 5.5 (Figure S18C), reflecting the titrations presented in Figure 3 of Senger *et al.*.[10] These figures were generated using the model shown in Figure S18D in which H_{ox} and H_{ox}-DT_i undergo a simple one-electron reduction to give H_{red} and H_{red}-DT_i, respectively. Meanwhile, H_{ox} and H_{red} convert to H_{ox}-DT_i and H_{red}-DT_i by simple binding of SO_2. As the concentration of SO_2 depends on pH, the SO_2 bound states are more abundant in the pH 5.5 titration than in the pH 6.5 and pH 7.5 titrations. Furthermore, as the redox potential for the H_{ox}-DT_i/H_{red}-DT_i transition is more positive than the H_{ox}/H_{red} transition, the apparent redox midpoint potentials of all the titration curves shift with pH, having a value of ≈ -350 mV at pH 7.5, ≈ -320 mV at pH 6.5, and ≈ -300 mV at pH 5.5. Importantly, while the redox potentials for the H_{ox}/H_{red} and H_{ox}-DT_i/H_{red}-DT_i transitions were explicitly defined in the model to be pH-independent, the apparent redox potential for the same transitions appear to be pH-dependent in Figures S18A-C. This is simply the consequence of the coupled equilibria (Figure S18D).

A further curiosity of the results from Senger *et al.* is that, as they titrate the hydrogenase from high potential to low potential at pH 6.5 and pH 5.5, they change from a mixture of H_{ox}/H_{ox}-DT_i to mostly H_{red}-DT_i and in a further step to H_{red} (see Figure 3 in ref.[10]), suggesting that H_{red}-DT_i conversion to H_{red} requires reduction. It is important to note that in this experiment the researchers apply an electrochemical potential while flushing gas that has been bubbled through a solution containing NaDT. Our explanation of these results is that at high potentials, NaDT will be oxidized by the electrode generating SO_2, which can bind to H_{ox} forming H_{ox}-DT_i. As the potential becomes more negative, H_{ox}-DT_i is converted to H_{red}-DT_i, but at the same time NaDT stops being oxidized to SO_2 and the SO_2 that was produced at high potentials is purged from the protein film because of the constant gas flow. This decreases the SO_2 concentration to zero, allowing H_{red}-DT_i to covert to back to H_{red}. This behavior is not easily described by a simple thermodynamic model and as such is not observed in Figure S18. A final phenomenon from Senger *et al*. that we cannot yet explain is that they measure the redox potential for H_{ox}/H_{red} at pH 7.5 to be ≈ -450 mV. Multiple publications from our laboratory have estimated this redox potential to be ≈ -350 mV using both spectroelectrochemical IR titrations[19, 22] and pH titrations under H_2[19] (also in this study).

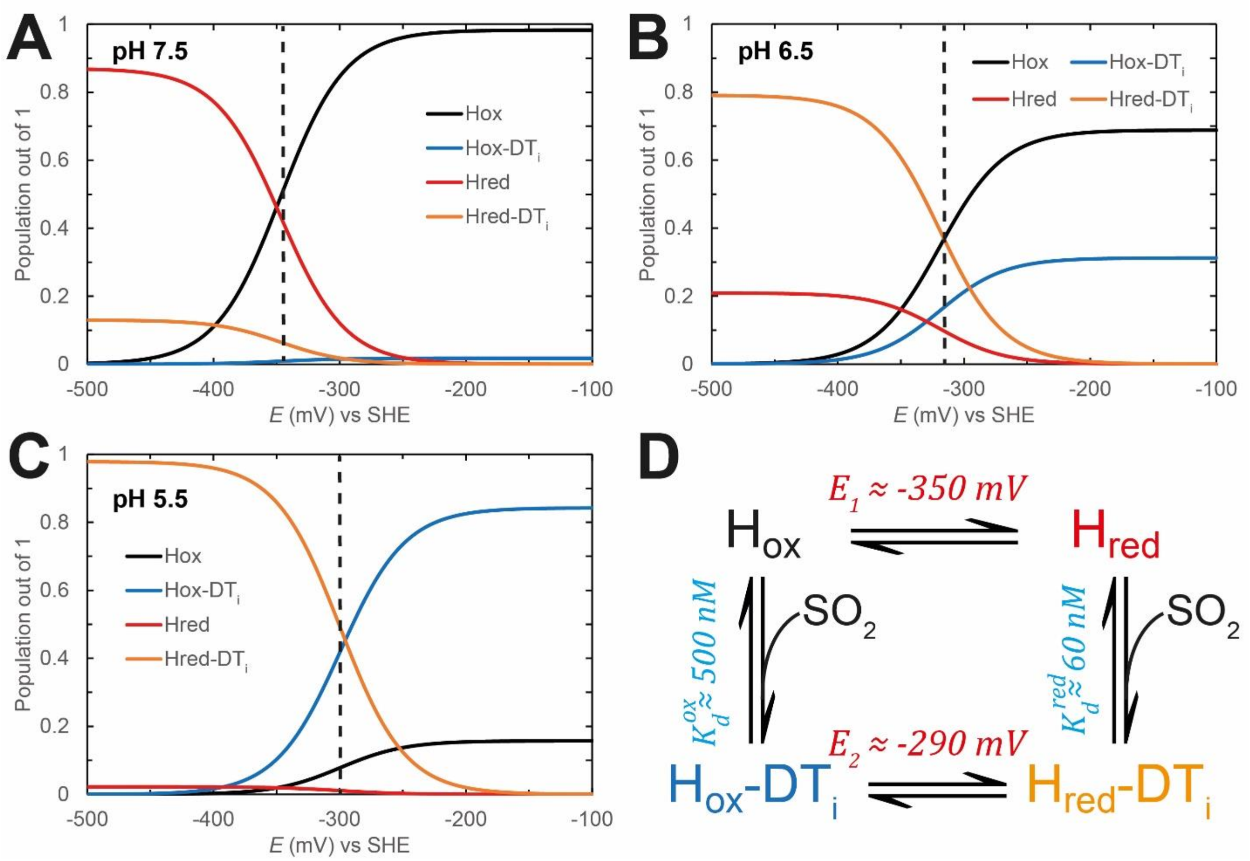

Figure S18 – Model for pH dependent redox potential titrations of *Cr*HydA1[PDT]. The variation in the populations of the H_{ox}, H_{red}, H_{ox}-DT_i and H_{red}-DT_i states (out of a total of 1) with the applied potential at pH 7.5 (**A**), 6.5 (**B**) and 5.5 (**C**) are shown based on equations derived using the model shown in **D**. H_{ox} and H_{red} are connected by a one electron reduction with a redox potential (E_1) of -350 mV, H_{ox}-DT_i and H_{red}-DT_i are connected by a one electron reduction with a redox potential (E_2) of -290 mV, H_{ox} and H_{ox}-DT_i are connected by SO_2 binding with a dissociation constant (K_d^{ox}) of 500 nM, and H_{red} and H_{red}-DT_i are connected by SO_2 binding with a dissociation constant (K_d^{red}) of 60 nM (this work). The SO_2 concentration was calculated using the pH-dependent speciation of Na_2SO_3 based on a *pKa* value for $SO_3^{2-} + H^+ \rightleftharpoons HSO_3^-$ of 7.19 and equilibrium constant for $HSO_3^- + H^+ \rightleftharpoons SO_2 + H_2O$ of 1.79,[23] setting the total concentration of $[Na_2SO_3] = [SO_3^{2-}]+[HSO_3^-]+[SO_2] = 10$ mM (in Senger *et al.*[10], 10 mM NaDT was present in the assay). The dotted lines indicate the midpoint potentials of the titration curves (i.e. the position where each state is at 50% of its maximum population) at each pH value.

Supplementary References

1. Kuchenreuther, J. M.; Grady-Smith, C. S.; Bingham, A. S.; George, S. J.; Cramer, S. P.; Swartz, J. R., High-yield expression of heterologous [FeFe] hydrogenases in *Escherichia coli*. *PLoS One* **2010,** *5* (11), e15491.
2. Esselborn, J.; Lambertz, C.; Adamska-Venkatesh, A.; Simmons, T.; Berggren, G.; Noth, J.; Siebel, J.; Hemschemeier, A.; Artero, V.; Reijerse, E.; Fontecave, M.; Lubitz, W.; Happe, T., Spontaneous activation of [FeFe]-hydrogenases by an inorganic [2Fe] active site mimic. *Nat. Chem. Biol.* **2013,** *9* (10), 607-609.
3. Birrell, J. A.; Wrede, K.; Pawlak, K.; Rodriguez-Maciá, P.; Rüdiger, O.; Reijerse, E. J.; Lubitz, W., Artificial Maturation of the Highly Active Heterodimeric [FeFe] Hydrogenase from Desulfovibrio desulfuricans ATCC 7757. *Isr. J. Chem.* **2016,** *56* (9-10), 852-863.
4. Le Cloirec, A.; C. Davies, S.; J. Evans, D.; L. Hughes, D.; J. Pickett, C.; P. Best, S.; Borg, S., A di-iron dithiolate possessing structural elements of the carbonyl/cyanide sub-site of the H-centre of Fe-only hydrogenase. *Chem. Commun.* **1999,** (22), 2285-2286.
5. Li, H.; Rauchfuss, T. B., Iron carbonyl sulfides, formaldehyde, and amines condense to give the proposed azadithiolate cofactor of the Fe-only hydrogenases. *J. Am. Chem. Soc.* **2002,** *124* (5), 726-727.
6. Fourmond, V., QSoas: a versatile software for data analysis. *Anal. Chem.* **2016,** *88* (10), 5050-5052.
7. Wille, H. C.; Franz, H.; Röhlsberger, R.; Caliebe, W. A.; Dill, F. U., Nuclear resonant scattering at PETRA III : Brillant opportunities for nano – and extreme condition science. *J. Phys. Conf. Ser.* **2010,** *217*.
8. Rodríguez-Maciá, P.; Birrell, J. A.; Lubitz, W.; Rüdiger, O., Electrochemical investigations on the inactivation of the [FeFe] hydrogenase from *Desulfovibrio desulfuricans* by O_2 or light under hydrogen-producing conditions. *ChemPlusChem* **2017,** *82* (4), 540-545.
9. Rodriguez-Maciá, P.; Dutta, A.; Lubitz, W.; Shaw, W. J.; Rüdiger, O., Direct comparison of the performance of a bio-inspired synthetic nickel catalyst and a [NiFe]-hydrogenase, both covalently attached to electrodes. *Angew. Chem. Int. Ed.* **2015,** *54* (42), 12303-12307.
10. Senger, M.; Laun, K.; Wittkamp, F.; Duan, J.; Haumann, M.; Happe, T.; Winkler, M.; Apfel, U. P.; Stripp, S. T., Proton-coupled reduction of the catalytic [4Fe-4S] cluster in [FeFe]-hydrogenases. *Angew. Chem. Int. Ed.* **2017,** *56* (52), 16503-16506.
11. Senger, M.; Mebs, S.; Duan, J.; Shulenina, O.; Laun, K.; Kertess, L.; Wittkamp, F.; Apfel, U. P.; Happe, T.; Winkler, M.; Haumann, M.; Stripp, S. T., Protonation/reduction dynamics at the [4Fe-4S] cluster of the hydrogen-forming cofactor in [FeFe]-hydrogenases. *Phys. Chem. Chem. Phys.* **2018,** *20* (5), 3128-3140.
12. Winkler, M.; Senger, M.; Duan, J.; Esselborn, J.; Wittkamp, F.; Hofmann, E.; Apfel, U.-P.; Stripp, S. T.; Happe, T., Accumulating the hydride state in the catalytic cycle of [FeFe]-hydrogenases. *Nat. Commun.* **2017,** *8* (1), 16115.
13. Chernev, P.; Lambertz, C.; Brünje, A.; Leidel, N.; Sigfridsson, K. G. V.; Kositzki, R.; Hsieh, C.-H.; Yao, S.; Schiwon, R.; Driess, M.; Limberg, C.; Happe, T.; Haumann, M., Hydride binding to the active site of [FeFe]-hydrogenase. *Inorg. Chem.***2014,** *53* (22), 12164-12177.
14. Mebs, S.; Duan, J.; Wittkamp, F.; Stripp, S. T.; Happe, T.; Apfel, U.-P.; Winkler, M.; Haumann, M., Differential protonation at the catalytic six-iron cofactor of [FeFe]-hydrogenases revealed by ^{57}Fe nuclear resonance X-ray scattering and quantum mechanics/molecular mechanics analyses. *Inorg. Chem.* **2019,** *58* (6), 4000-4013.
15. Mebs, S.; Senger, M.; Duan, J.; Wittkamp, F.; Apfel, U.-P.; Happe, T.; Winkler, M.; Stripp, S. T.; Haumann, M., Bridging hydride at reduced H-cluster species in [FeFe]-hydrogenases revealed by infrared spectroscopy, isotope editing, and quantum chemistry. *J. Am. Chem. Soc.* **2017,** *139* (35), 12157-12160.
16. Birrell, J. A.; Pelmenschikov, V.; Mishra, N.; Wang, H.; Yoda, Y.; Tamasaku, K.; Rauchfuss, T. B.; Cramer, S. P.; Lubitz, W.; DeBeer, S., Spectroscopic and computational evidence that [FeFe]

hydrogenases operate exclusively with CO-bridged intermediates. *J. Am. Chem. Soc.* **2020,** *142* (1), 222-232.

17. Sanchez, M. L. K.; Sommer, C.; Reijerse, E.; Birrell, J. A.; Lubitz, W.; Dyer, R. B., Investigating the kinetic competency of *Cr*HydA1 [FeFe] hydrogenase intermediate states via time-resolved infrared spectroscopy. *J. Am. Chem. Soc.* **2019,** *141* (40), 16064-16070.
18. Lorent, C.; Katz, S.; Duan, J.; Kulka, C. J.; Caserta, G.; Teutloff, C.; Yadav, S.; Apfel, U.-P.; Winkler, M.; Happe, T.; Horch, M.; Zebger, I., Shedding light on proton and electron dynamics in [FeFe] hydrogenases. *J. Am. Chem. Soc.* **2020,** *142* (12), 5493-5497.
19. Rodríguez-Maciá, P.; Breuer, N.; DeBeer, S.; Birrell, J. A., Insight into the redox behavior of the [4Fe–4S] subcluster in [FeFe] hydrogenases. *ACS Catal.* **2020,** *10* (21), 13084-13095.
20. Haumann, M.; Stripp, S. T., The molecular proceedings of biological hydrogen turnover. *Acc. Chem. Res.* **2018,** *51* (8), 1755-1763.
21. Zhang, Z.; Ewing, G. E., Infrared spectroscopy of SO_2 aqueous solutions. *Spectrochim. Acta A Mol. Biomol. Spectrosc.* **2002,** *58* (10), 2105-2113.
22. Adamska-Venkatesh, A.; Krawietz, D.; Siebel, J.; Weber, K.; Happe, T.; Reijerse, E.; Lubitz, W., New redox states observed in [FeFe] hydrogenases reveal redox coupling within the H-cluster. *J. Am. Chem. Soc.* **2014,** *136* (32), 11339-11346.
23. Selwyn, L.; Tse, S., The chemistry of sodium dithionite and its use in conservation. *Stud. Conserv.* **2008,** *53* (sup2), 61-73.
